# MicroRNA Signature in Melanoma: Biomarkers and Therapeutic Targets

**DOI:** 10.3389/fonc.2021.608987

**Published:** 2021-04-22

**Authors:** Soudeh Ghafouri-Fard, Mahdi Gholipour, Mohammad Taheri

**Affiliations:** ^1^ Department of Medical Genetics, Shahid Beheshti University of Medical Sciences, Tehran, Iran; ^2^ Urology and Nephrology Research Center, Shahid Beheshti University of Medical Sciences, Tehran, Iran

**Keywords:** miRNA, melanoma, biomarker, expression, polymorphism

## Abstract

Melanoma is the utmost fatal kind of skin neoplasms. Molecular changes occurring during the pathogenic processes of initiation and progression of melanoma are diverse and include activating mutations in BRAF and NRAS genes, hyper-activation of PI3K/AKT pathway, inactivation of p53 and alterations in CDK4/CDKN2A axis. Moreover, several miRNAs have been identified to be implicated in the biology of melanoma through modulation of expression of genes being involved in these pathways. In the current review, we provide a summary of the bulk of information about the role of miRNAs in the pathobiology of melanoma, their possible application as biomarkers and their emerging role as therapeutic targets for this kind of skin cancer.

## Introduction

Arising from unrestrained proliferation of melanocytes, melanoma is the utmost fatal kind of skin neoplasm (1). Though melanoma encompasses less than 5% of all skin cancers, it accounts for most of skin neoplasms mortalities (2). When the cancer is diagnosed in early stages, surgical resection of the tumor is the appropriate therapeutic options for enhancement of survival of patients. Yet, based on the metastatic potential of melanoma, surgery is not satisfactory in advanced stages of melanoma (3). Although the mortality rate of primary melanoma is about 11%, metastatic melanoma has a poor prognosis resulting from inefficiency of conventional therapies (4, 5). Meanwhile, novel therapeutic option might offer efficient methods for these patients. For instance, immunotherapeutic approaches such as administration of Anti-PD1 (nivolumab, pembrolizumab) alone, or the combination of anti-PD1 with anti-cytotoxic T lymphocyte-associated protein 4 (CTLA4) ipilimumab has raised the survival of patients who suffer from advanced stages of melanoma (6).

Targeted therapies, like combinations of BRAF inhibitors (Dabrafenib) and MEK inhibitors (vemurafenib) are also frequently used on BRAFV600E mutant melanomas. Superficial spreading, nodular, lentigo maligna and acral lentiginous melanomas represent the main types of melanoma with the first one being the most frequent type (4). Ultraviolet radiation and melanocytic nevi are two main risk factors for development of this kind of skin cancer (4). Molecular changes occurring during the pathogenic processes of initiation and progression of melanoma are diverse and include activating mutations in BRAF and NRAS genes, hyper-activation of PI3K/AKT pathway, inactivation of p53 and alterations in CDK4/CDKN2A axis (4). In addition, several studies have shown the critical role of microRNAs (miRNAs) both in the initiation and in the progression of melanoma (7). These transcripts have sizes around 22 nucleotides and are generated through a multi-step process from DNA sequences into primary, precursor and mature miRNAs, respectively. As a general rule, they regulate gene expression through binding with complementary sequences in the 3′ untranslated region (3′ UTR) of mRNAs and subsequently lead to degradation and suppression of translation of the target transcript. Less frequently, they interact with the 5′ UTR, coding or promoter regions (8). Moreover, there are some reports of activation of translation of certain genes by miRNAs in some situations. For instance, let-7 family of miRNAs can induce translation when cell cycle is arrested in spite of their inhibitory effects on translation during cell proliferation (9). Therefore, miRNAs are regarded as important mediators of gene expression. Besides, their presence in extracellular vesicles provides them the opportunity to module communication between various cells (8). In the current paper, we summarize the bulk of information about the role of miRNAs in the pathobiology of melanoma, their possible application as biomarkers and their emerging role as therapeutic targets for this kind of skin cancer.

## Dysregulated miRNAs in Melanoma

Expression pattern of miRNAs in melanoma cell lines and clinical specimens has been assessed by both high throughput and candidate gene approaches. An example of the former types of studies is the study conducted by Zhang et al. (10). They reported DNA copy number changes in miRNA coding genes in the majority of the assessed melanoma samples. Notably, miRNA copy alterations have been correlated with miRNA expression. Moreover, they reported copy number alterations in genes contributing in the biogenesis or function of miRNAs in tumor samples (10). Through a microarray-based technique, Aksenenko et al. have identified differential expression of 143 miRNAs between melanoma samples and adjacent skin tissues. Among the dysregulated miRNAs has been the up-regulated miRNA hsa-miR-146a-5p which has been predicted to be associated with Toll-like receptor, NF-κB and ErB pathways. Moreover, this miRNA has been shown to target one of the most recurrently mutated genes in melanoma i.e., the NRAS gene (11).

**Figure 1:**
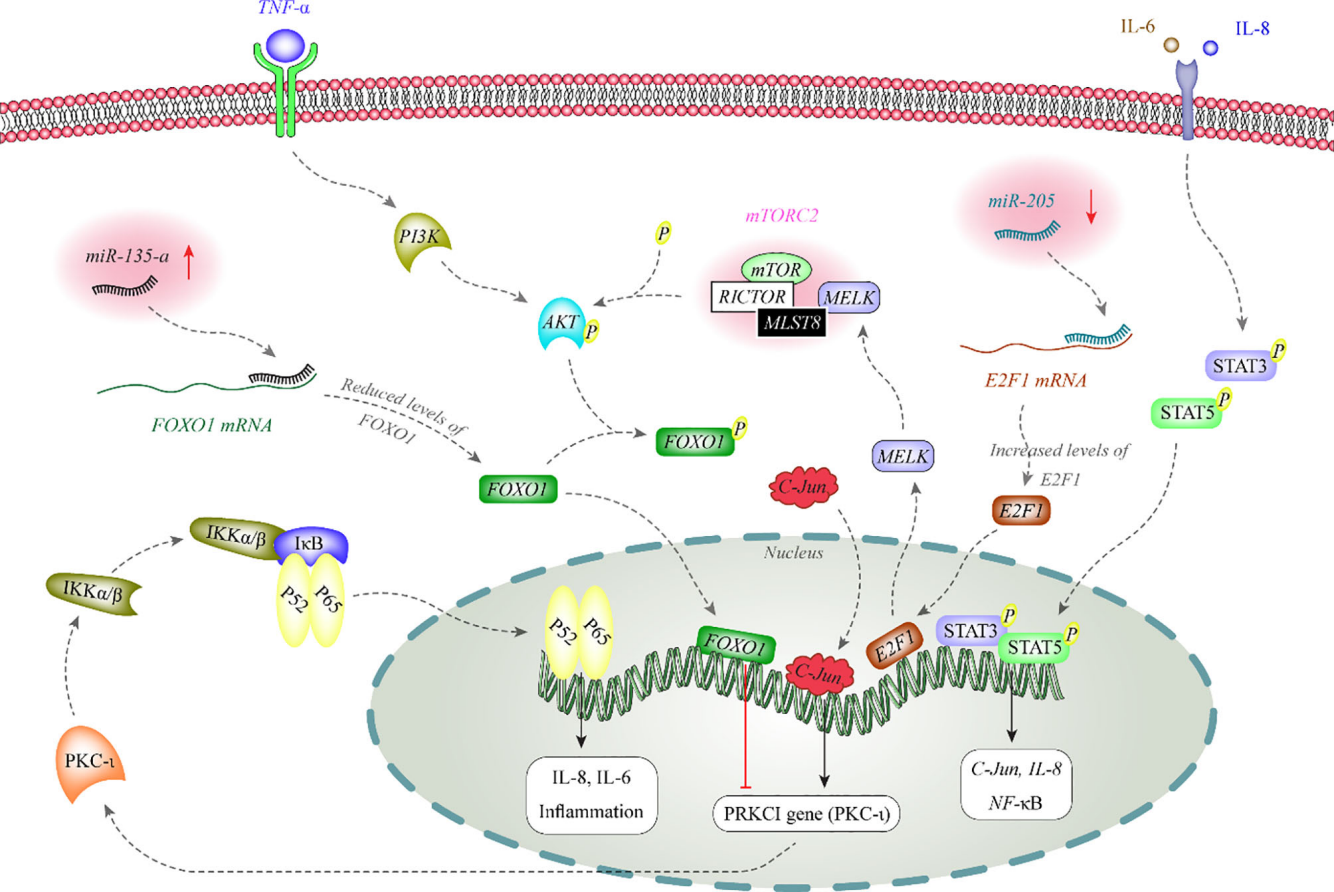
AKT phosphorylates FOXO1 to inhibit its nuclear translocation. FOXO1 has a role in the suppression of expression of PKC-iota in the nucleus. PKC-iota is an inducer of NF-κB which enhances expression of inflammatory genes in the nucleus. Expression of miR-135-a is increased in melanoma. This miRNA binds with the 3’ UTR of FOXO1 to decrease its expression (12, 13). On the other hand, miR-205 is decreased in melanoma. This miRNA inhibits expression of E2F1 through binding with its 3’ UTR. E2F1 increases expression of MELK. MELK activates mTORC2 through binding with MLST8. mTORC2 has a role in phosphorylation and activation of AKT (14).

miRNA also affect activity of melanoma-related signaling pathways. **Figure 1** depicts the functional association between two miRNAs and AKT and NF-κB signaling pathways.

Expression profiling of miRNAs in melanocytes and melanoma cells originated from primary or metastatic melanoma cells has provided valuable data about the role of miRNAs in each phase of cancer development. A panel of miRNAs including miR-133a, miR-199b, miR-453, miR-520f, miR-521, and miR-551b has been found consistently up-regulated in the course of cancer development from melanocytes to primary cancerous cell and from primary to metastatic melanomas. On the other hand, miR-190 had the opposite trend during this course. Furthermore, expressions of miR-126, miR-29c, miR-506, miR-507, and miR-520d* have been found to be increased during the early progression of melanoma and have been decreased in the metastatic phase. Two other miRNAs including miR-489 and miR-527 had the opposite pattern of expression (15). Levati et al. have demonstrated up-regulation of miR-17-5p, miR-18a, miR-20a, and miR-92a while down-regulation of miR-146a, miR-146b and miR-155 in most of assessed melanoma cell lines compared with melanocytes (16).

Other studies have reported dysregulation of several other miRNAs in the melanoma samples. Among up-regulated miRNAs are miR-221 and miR-222 which induce malignant features through decreasing expression of c-KIT receptor and p27Kip. Both miRNAs promote epithelial-mesenchymal transition (17, 18). Moreover, expression of miR-210 has been demonstrated to be elevated in several cancer types including melanoma. Its expression has been correlated with metastatic potential of melanoma tumors. Up-regulation of miR-210 in cancer cell lines facilitates evasion from hypoxia-induced cell cycle arrest and partly upturned the hypoxic gene expression profile. This miRNA has been revealed to target a known MYC antagonist namely MNT. Therefore, miR-210 has been shown to modulate the hypoxia response in cancer cells *via* regulating an important transcriptional suppressor of the MYC-MAX axis (19). In an attempt to detect the miRNAs that are regulated by BRAFV600E mutation *via* the ERK pathway, Vitiello et al. have conducted RNA sequencing on A375 cell line and a vemurafenib-resistant clone. Their experiments have led to identification of miR-204 and miR-211 as the utmost over-expressed miRNAs by vemurafenib. In spite of belonging to an identical miRNA family, miR-204 and miR-211 have distinguishing characteristics. miR-204 is regulated by STAT3 and its transcript levels are increased in amelanotic melanoma cells, where it functions as a mediator of anti-migratory effects of vemurafenib by modulating expression of AP1S2. On the contrary, miR-211, as a direct target of MITF, is over-expressed in melanotic melanoma cells. miR-211 regulates expression of EDEM1 and subsequently weakens the destruction of Tyrosinase. Thus, miR-211 is a facilitator of pro-pigmentation function of vemurafenib (20). **Table 1** displays the list of over-expressed miRNAs in melanoma.

**Table 1:** List of over-expressed miRNAs in melanoma.

microRNA	Samples	Assessed cell lines	Functional analysis	Gene interaction	Signaling pathway	Association with clinical features	Function	Reference
*miR-211*	SCID mice (SKMEL28 or SK-P8-2 or 501-Mel and 501-Mel-P5-5 cell lines were injected to mice)	SKMEL28, vemurafenib-resistant SKMEL28 and 501-Mel cell lines	Yes	–	PI3K signaling pathway	–	Has oncogenic role. Its deletion attenuates proliferation, invasion and tumorigenicity and inhibits PI3K signaling. Also induces metabolic vulnerability of melanoma cells and sensitizes vemurafenib resistant cells to vemurafenib	(21)
*miR-211-5p*	NOD/SCID/IL2gR^-/-^ (NSG) mice (A375 cell line was injected to mice)	A375, SK-Mel-103, SK-Mel-28, SK-Mel-147	Yes	NUAK1, SLUG	–	–	Promotes proliferation and induces resistance to vemurafenib and MEK inhibitor trametinib in melanoma cells	(22)
*miR-211-5p*	86 melanoma tissues, serum samples from 130 healthy controls and 255 melanoma patients	–	No	–	–	Disease stage, survival	A possible diagnostic biomarker	(23)
*miR-16*	86 melanoma tissues, serum samples from 130 healthy controls and 255 melanoma patients	–	No	–	–	Disease stage	A possible diagnostic biomarker	(23)
*miR-204-5p*	NOD/SCID/IL2gR^-/-^ (NSG) mice (A375 cell line was injected to mice)	A375, SK-Mel-103, SK-Mel-28, SK-Mel-147	Yes	EFNB2, NUAK1, SLUG	–	–	Promotes proliferation and induces resistance to vemurafenib and MEK inhibitor trametinib in melanoma cells	(22)
*miR-378*	36 melanoma tissues and paired ANTs, 14 Nude athymic BalB/C mice (A875 cell line was injected to mice)	A875, A375	Yes	FOXN3	Wnt/β-catenin signaling pathway	lymph node metastasis	Induces migration and invasion and activates EMT process in melanoma cells through downregulation of FOXN3 and activation of Wnt/β-catenin pathway	(24)
*miR-378a-5p*	FFPE tissues specimens of 27 metastatic melanoma and 13 *in situ* melanoma, female mice (M14 cell line was injected to mice)	M14, A375, SBCL1, HUVEC	Yes	STAMBP, HOXD10	–	–	Enhances migration, invasion and angiogenesis ability of melanoma cells	(25)
*miR-1908*	71 paraffin-embedded melanoma skin lesions, NOD scid, NOD scid gamma, athymic nu/nu, and C57Bl6 mice (MeWo-LM2 cell line was injected to mice)	MeWo-LM2, A375, SK-Mel-2, WM-266-4, HT-144, A2058, HUVECs	Yes	ApoE, DNAJA4	ApoE signaling	shorter metastasis-free survival	Augments invasion, metastasis, metastatic endothelial recruitment (MER) and angiogenesis in melanoma cells through targeting ApoE and DNAJA4	(26)
*miR-199a-3p*			Yes	ApoE, DNAJA4	ApoE signaling	shorter metastasis-free survival		(26)
*miR-199a-5p*			Yes	ApoE, DNAJA4	ApoE signaling	shorter metastasis-free survival		(26)
*miR-106b*	97 primary cutaneous melanoma tissue samples, 17 melanoma metastases, 15 dysplastic nevi	–	No	–	–	Poor prognosis, Breslow thickness, tumor ulceration, advanced clinical stage	May implicate in progression of cutaneous melanoma and can be a potential prognostic biomarker	(27)
*miR-106a*	–	A375, A2058, HEMn,	Yes	Cx43	–	–	Enhances melanoma cells proliferation *via* suppression of Cx43	(28)
*miR-146a*	FFPE tissue specimens of 22 primary melanoma tumors, 18 nevocellular nevi, 13 healthy skin samples, wild type and miR146a-/- C57BL/6 mice (B16.F10 cell line was injected to mice)	B16.F10	Yes	Stat1	–	TNM stage	Negatively regulates immune responses. also affects proliferation, migration and mitochondrial fitness of melanoma cells through regulating STAT1/IFNγ axis	(29)
*miR-146a*	Mice (A375 cell line was injected to mice)	A375, MA-1, MC-1, MA-2, MC-2, WK-Mel	Yes	LFNG, NUMB, ITGAV, ROCK1	NOTCH/PTEN/Akt pathway	–	Has dual function. It enhances melanoma cell growth but inhibits metastasis formation (and is poorly expressed in circulating tumor cells)	(30)
*miR-146a*	10 primary melanoma and nevus tissues from the same patients and 15 primary melanoma tissues and metastases from the same patients	WI-38, IMR-90t, 293T, SKMEL28	Yes	NUMB	Notch signaling pathway	–	Increases proliferative ability and tumorigenecity of melanoma cells through targeting NUMB	(31)
*miR-146a*	55 melanoma tissues and paired ANTs	A375, WM115, M14, G361, HACAT	Yes	SMAD4	–	TNM stage, lymph node metastasis	Promotes migration and invasion of melanoma cells through targeting SMAD4	(32)
*miR-10b*	FFPE tissue specimens of 40 primary melanomas that are metastasis-free, 39 primary melanomas with metastasis, 32 metastases	–	No	–	–	Tumor metastasis	Is a potential prognostic biomarker in detection of thicker melanomas that have enhanced risk of metastasis	(33)
*miR-10b*	–	Mel 505, PMWK, sk-mel-28, sk-mel-24, VMM39, MEL 224, YUHEF, YUROB,	Yes	–	–	–	Its expression positively correlates with B-RafV600E mutation and increases anchorage-independent growth of B-Raf wild-type melanoma cells	(34)
*miR-10b*	78 melanoma tissues and 30 non-tumor skin samples, nude mice (A375 cell line was injected to mice)	A375, SK-MEL-1, SK-MEL-28, WM451, human primary melanocytes	Yes	ITCH	Wnt/β-catenin signaling pathway	Overall survival	Its knockdown results in ITCH-mediated suppression of proliferation, migration and invasion in melanoma cells.	(35)
*miR-21*	67 malignant melanoma tissue and 67 normal control skin samples	–	No	PDCD4	–	tumor size, higher Clark classification level, lymph node metastases	Can be a possible biomarker or therapeutic target in melanoma	(36)
*miR-21*	86 primary cutaneous melanomas tissues, 10 melanoma metastases, 10 dysplastic nevi samples	HTB-67, A375	Yes	–	–	Overall survival, Breslow thickness, advanced clinical stage,	Its silencing suppresses growth and increases apoptosis, chemosensitivity and radiosensitivity of melanoma cells	(37)
*miR-21*	12 FFPE primary melanoma tissues and 12 melanocytic nevi	WM9, WM35b, WM451, WM793, WM951, WM1205, SKMel23, SKMel113, MV3, MEWO	Yes	Cdc25a	–	Recurrence-free survival, overall survival	Its downregulation promotes apoptosis.	(38)
*miR-21*	female 01B74 Athymic NCr-nu/nu mice (A375 cell line was injected to mice)	WM1552c, WM793b, MEL 39, A375	Yes	TIMP3	–	–	Increases invasive ability of melanoma cells through targeting TIMP3	(39)
*miR-21*	45 melanoma tissues and ANTs	A375	Yes	SPRY1, PDCD4, PTEN	ERK/NF-κB signaling pathway	histological differentiation, TNM stage, lymphatic metastasis	Its inhibition decreases proliferation, migration and invasion and induces apoptosis	(40)
*miR-21*	BALB/c nude mice (OCM-1 cell line was injected to mice)	OCM-1, M619, MuM-2B	Yes	p53	–	–	Promotes proliferation, migration and invasion of melanoma cells through targeting p53	(41)
*miR-21-5p*	20 melanoma tissues and paired ANTs	A375, M14	Yes	CDKN2C	–	–	Enhances proliferation and cell cycle G1/S transition in melanoma cells through targeting CDKN2C	(42)
*miR-652*	26 uveal melanoma tissues and paired ANTs	MUM-2B, MEL270, ARPE-19	Yes	HOXA9	HIF-1alpha signaling	–	Increases proliferation and migration in uveal melanoma cell through promoting HIF-1alpha signaling by suppression of HOXA9	(43)
*miR-367*	28 uveal melanoma tissues and paired ANTs	M17, M23, MUM-2B, C918, um95	Yes	PTEN	–	–	Enhances proliferation and migration in uveal melanoma cell *via* targeting PTEN	(44)
*miR-4286*	FFPE specimens of 16 melanoma tissues and 3 melanocytic nevi samples	BRO, SK-MEL-1	Yes	APLN, FPGS, GPR55, HMGA1, RRN3, TP523	–	–	Its inhibition results in decreased proliferation and increased apoptosis.	(45)
*miR-367*	50 melanoma tissues and 25 benign nevi tissues, 6 Nude mice (A375 cell line was injected to mice)	A375, WM35, SK-MEL-5, SK-MEL-2, HEMa-LP	Yes	PTEN	–	Decreased overall survival, tumor thickness, TNM stage, lymph node involvement, distant metastasis	Elevates proliferation, migration and invasion in cutaneous melanoma cells through targeting PTEN	(46)
*miR-638*	7 primary melanomas, 9 lymph node metastases, and 8 remote skin metastases	BRO, A-375, HT144, RPM-MC, 1F6, HEM, SK-Mel-147, SK-Mel-28	Yes	TP53INP2	p53 signaling pathway	–	Enhances proliferation and invasion of melanoma cells and prevents apoptosis and autophagy *via* targeting TP53INP2	(47)
*miR-338-5p*	46 melanoma tissues and 25 normal nevi samples, Nude mice (A375 cell line was injected to mice)	A375, WM35, SK-MEL-5, SK-MEL-2, HEMa-LP	Yes	CD82	AKT pathway	Poor Prognosis, patients survival, tumor stage, metastasis	Promotes proliferation and metastasis *via* targeting CD82	(48)
*miR-363-3p*	–	A2058, WM793B	Yes	p21	–	–	Promotes stemness of melanoma cells *via* suppression of p21	(49)
*miR-15b*	128 FFPE tissues of primary melanomas and 11 melanocytic nevi samples	WM9, WM35, WM451, WM793, WM951, WM1205, SKMel23, SKMel113, MV3, MeWo	Yes	–	–	Overall survival	Its knockdown decreases proliferation and induces apoptosis.	(50)
*miR-454*	25 uveal melanoma tissues and ANTs	OCM‐1A, MUM‐2C, C918, MUM‐2B, D78	Yes	PTEN	–	–	Promotes cell proliferation, colony formation and invasion uveal melanoma cells	(51)
*miR-214*	57 primary melanoma tissues, 13 *in situ* melanomas and 18 cutaneous metastases, female CD1 nude mice (A375 or 106 WK‐Mel, GR4‐Mel, 1300‐Mel, SK‐Mel‐173 and SK‐Mel‐197 cell lines were injected to mice)	293T, MDA-MB-231, 4T1, A375, 1300-Mel, GR4-Mel, WK-Mel, Dett-Mel, SK-MEL-103, SK-MEL-173, SK-MEL-187, SK-MEL-197, HEMa-LP	Yes	TFAP2C	–	–	Enhances cell movement and metastasis *via* suppression of TFAP2C	(52)
*miR-122-5p*	Human melanoma tissues and pigmented nevus tissues	293T, SK-MEL-110, A375	Yes	NOP14	–	–	Its inhibition represses proliferation and induces cell cycle arrest at G1 phases through regulation of NOP14	(53)
*miR-182*	22 primary melanoma tissues, 59 metastatic melanoma tissues and 19 nevi samples, C57BL/6J mice (B16F10 cell line was injected to mice)	SK-MEL-19, SK-MEL-29, SK-MEL-85, SK-MEL-94, SK-MEL-100, SK-MEL-103, SK-MEL-147, SK-MEL-173, SK-MEL-187, SK-MEL-192, SK-MEL-197, 501mel, HEK293T, A375, B16F10, WM35	Yes	FOXO3, MITF-M	–	–	Enhances migration, invasion and metastasis in melanoma cells through suppression of FOXO3 and MITF-M expression	(54)
*miR-221*	Serum samples from 72 cutaneous malignant melanoma and 54 healthy controls	–	No	–	–	Patient survival, tumor thickness, differentiation, T classification, N classification, metastasis, advanced clinical stage	Can be a potential prognostic biomarker in cutaneous melanoma	(55)
*miR-221*	–	WM35, WM983A, WM164, 1205Lu	Yes	cKit, p27 (Kip1)	–	–	Promotes proliferation of melanoma cells through targeting cKit and p27. Also its inhibition induces apoptosis	(18)
*miR-767*	8 melanoma tissues and ANTs	MeWo, MHEM, A375, WM-115, UACC257, WM35, A7, PEM	Yes	CYLD	–	–	Enhances proliferation of melanoma cells through inhibition of CYLD	(56)
*miR-135a*	20 melanoma tissues and paired ANTs	HEM, sk-mel-1, A375	Yes	FOXO1	AKT signaling pathway	–	Promotes melanoma cells proliferation, tumorigenicity and cell cycle progression *via* targeting FOXO1	(12)
*miR-135b*	20 melanoma tissues and paired ANTs	A-375	Yes	LATS2	–	–	Its inhibition decreases proliferation and migration and induces apoptosis in melanoma cells	(57)
*miR-25*	–	A875, MV3, M14, uacc-257, HEM-a	Yes	RBM47	PI3K/Akt/mTOR signaling pathway	–	Promotes proliferation and migration of melanoma cells through targeting RBM47	(58)
*miR-25*	30 primary melanoma tissues and related non-cancerous skin samples	HEM, MV3, SK-HEP-1, A375	Yes	DKK3	WNT/β-Catenin signaling Pathway	–	Enhances proliferation and invasion in melanoma cells *via* targeting DKK3	(59)
*miR-125a*	22 melanoma tissue	SK-MEL-239, A375, 451Lu	Yes	BAK1, MLK3	–	–	Promotes BRAF inhibitors resistance through inhibition of intrinsic apoptotic pathway by targeting BAK1 and MLK3	(60)
*miR−106b−5p*	18 primary melanoma tissues and 18 benign nevi	SK-MEL-1, A-375, HEM	Yes	PTEN	Akt/ERK signaling pathway	–	Promotes proliferation and progression of melanoma through targeting PTEN and regulation of Akt/ERK pathway	(61)
*miR-181b*	3 uveal melanoma tissues and 3 normal tissues	SP6.5, VUP, OCM1, 92-1, MUM2b	Yes	CTDSPL	–	–	Promotes cell cycle progression in uveal melanoma cells through targeting CTDSPL	(62)
*miR-769*	8 melanoma tissues and ANTs	g MHEM, SK-MEL-28, WM-115, UACC257, A375, A7, MeWo, PEM	Yes	GSK3B	–	–	Enhances proliferation of melanoma cells *via* targeting GSK3B and suppression of its expression	(63)
*miR−20a*	10 uveal melanoma tissues and 10 normal uveal tissues	MUM-2B, MUM-2C, D78	Yes	–	–	–	Promotes proliferation, migration and invasion in uveal melanoma cells	(64)
*miR-30d*	109 primary melanoma tissues and 17 melanoma metastases	HEK293T, A375, B16F10, WM35, WM98	Yes	GALNT7	–	Overall survival, tumor thickness, tumor stage, shorter time to recurrence	Promotes metastatic capacity of melanoma cells through targeting GALNT7	(65)
*miR-30b*	109 primary melanoma tissues and 17 melanoma metastases	HEK293T, A375, B16F10, WM35, WM98	Yes	–	–	Overall survival, tumor thickness, tumor stage, shorter time to recurrence	Promotes metastatic capacity of melanoma cells	(65)
*miR-224*	Primary melanoma tissues and melanoma metastases, athymic NMRI nude mice	SK-Mel-28, SK-Mel-29, SK-Mel-103, SK-Mel-147	Yes	TXNIP	–	–	Increases migration and invasion and induces EMT process through targeting TXNIP	(66)
*miR-452*	Primary melanoma tissues and melanoma metastases, athymic NMRI nude mice	SK-Mel-28, SK-Mel-29, SK-Mel-103, SK-Mel-147	Yes	TXNIP	–	–	Increases migration and invasion and induces EMT process through targeting TXNIP	(66)
*miR-19b*	14 melanoma tissues, C57BL/6 mice	293T, A2058, CRL1579, SKMEL28, G361, HNEM	Yes	PITX1	–	–	Regulates proliferation and hTERT expression in melanoma cells through targeting PITX1	(67)
*miR-3151*	21 RNA samples of melanoma patients	MM A375, Mel-39, MeWo, HEK293, A375	Yes	TP53	–	–	Its knockdown induces TP53-mediated inhibition of proliferation and promotion of apoptosis in melanoma cells	(68)
*miR-301a*	46 melanoma tissues and 18 benign melanocytic naevi	SK-MEL-1, A-375	Yes	PTEN	Akt and FAK signaling pathways	Poor prognosis, metastasis	Its inhibition suppresses proliferation, colony formation, migration and invasion in melanoma cells through targeting PTEN.	(69)
*miR-4262*	110 cutaneous melanoma tissues and ANTs	HACAT, HFF, A375, Malme-3M, SK-MEL-2, SK-MEL-5, M14	Yes	KLF6	–	–	Promotes proliferation of melanoma cells through targeting KLF6	(70)
*miRNA-106b*	Female athymic nude mice (A375 cell line was injected to mice)	A375, Hs294t, SK-Mel28, SK-Mel 119, Mel 1241, Mel 1011, Mel 928, NHEM	Yes	–	–	–	Its downregulation inhibits melanoma cells proliferation and induces cell cycle arrest at G1 phase	(71)
*miR-519d*	21 primary melanoma tissues, 19 normal skin and 21 metastatic melanoma samples, C.B-17/Icr-scid mice (A2058 cell line was injected to mice)	A2058, SK-Mel-28, A375, SK-Mel-90, MeWo	Yes	EphA4	ERK1/2 signaling pathway	–	Promotes proliferation, migration and invasion of melanoma cells *via* downregulation of EphA4	(72)
*miR-370*	41 melanoma tissues and ANTs, BALB/c nude mice (A375 cell line was injected to mice)	SK-MEL-1, A375, HEMn-LP	Yes	PDHB	–	TNM stage	Promotes proliferation, invasion and glycolysis in melanoma cells and induces apoptosis through targeting PDHB	(73)
*miR-373*	16 melanoma tissues and normal skin samples	A375, WM115, WM75, mela	Yes	SIK1	–	–	Promotes migration of melanoma cells through targeting SIK1	(74)
*miR-92a*	75 melanoma tissues and paired ANTs	A375.S2, A7, MeWo, RPMI-7951, SK-MEL-5, SK-MEL-24, SKMEL-28, PEM	Yes	–	–	Overall survival, tumor stage, lymph node metastasis, distant metastasis	Its knockdown suppresses proliferation and migration of melanoma cells	(75)
*miR-517*	62 melanoma tissues and 40 normal skin tissues	HACAT, A375, G-361, OCM-1	Yes	CDKN1C	JNK signaling pathway	–	Its silencing induces oxidative stress injury in melanoma cells through upregulation of CDKN1C and inactivation of JNK signaling pathway	(76)
*miR-27a*	43 paraffin‐embedded melanoma tissues and 22 pigmented nevus samples, female BALB/c nude mice (A375 cell line was injected to mice)	Mel‐RM, A375	Yes	SYK	mTOR signaling pathway	TNM staging, lymph node metastasis	Its silencing promotes autophagy and apoptosis in melanoma cells through SYK-mediated modulation of mTOR signaling pathway	(77)
*miR-186*	8 melanoma tissues and ANTs	A375-S2, SKMEL-28, SKMEL-5, MeWO, RPMI-7951, NHEM	Yes	CYLD	–	–	Enhances proliferation and anchorage-independent growth of melanoma cells *via* downregulation of CYLD	(78)
*miR-1246*	Tissues from 43 melanoma patients	HEM, A375, A2058	Yes	FOXA2	–	–	promoted cell viability and metastasis in melanoma cells *via* targeting FOXA2	(79)
*miR-150*	20 melanoma tissues and paired ANTs	M14, A357, WM115, NHEM	Yes	PDCD4	–	–	Its silencing inhibits cell proliferation, migration and invasion and induces apoptosis in melanoma cells.	(80)
*miR-520f*	10 melanoma and paired ANTs	UACC257, WM-115, A7, MeWo, A375, NHEM, WM-115, PEM	Yes	ITCH	–	–	Promotes proliferation, colony construction and anchorage-independent growth in melanoma cells *via* targeting ITCH	(81)
*miR−633*	11 melanoma tissues and 10 ANTs	A375, A2058, B16, MEL-RM and M21	Yes	KAI1	–	–	Raises migratory ability and proliferation of melanoma cells *via* targeting KAI1 and reducing KAI1 expression	(82)

Numerous tumor suppressor miRNAs have been down-regulated in melanoma samples. For instance, while miR-34a is constantly detected in normal melanocytes, it is not expressed in uveal melanoma cells. Forced over-expression of this miRNA in uveal melanoma cells remarkably diminishes their growth and migratory abilities. Mechanistically, this miRNA inhibits expression of c-Met protein and decreases the levels of phosphorylated Akt and cell cycle-related proteins (83). Besides, miR-34b, miR-34c, and miR-199a* have been shown to down-regulate MET expression, suppressing the invasive growth features in the melanoma cells (84). Furthermore, expressions of the let-7 miRNAs have been shown to be decreased in primary melanomas compared with benign nevus samples. Forced up-regulation of let-7b in melanoma cells has led to significant decrease in the expression of cyclins D1, D3, and A, and CDK4. The functional interaction between let-7b and cyclin D1 has been verified through *in vitro* experiments (85). The inhibitory effect of let-7a on expression of integrin beta 3 has been verified in another study (86). In addition, functional studies have shown the role of miR-155 in the suppression of proliferation of a number of melanoma cell lines and induction of apoptosis in these cells (16). **Table 2** lists the down-regulated miRNAs in melanoma.

**Table 2:** List of under-expressed miRNAs in melanoma.

microRNA	Samples	Assessed cell lines	Functional analysis	Gene interaction	Signaling pathway	Association with clinical features	Function	Reference
*miR-429*	6 BALB/c-nu mice (A375 cell line was injected to mice)	A-375, 293T	Yes	AKT1	–	–	Represses proliferation and migration of melanoma cells by targeting AKT1	(87)
*miR-429-5p*	55 melanoma tissues and normal skin tissues	A375, PEM	Yes	LIMK1	–	Tumor thickness, tumor stage	Blocks migration and invasion of melanoma cells through targeting LIMK1	(88)
*miRNA-326*	23 melanoma tissues and paired ANTs	SK-MEL-28, A375, HT144, A2058, HEMs	Yes	KRAS	AKT and ERK signaling pathways	–	Suppresses cell proliferation and invasion and promotes apoptosis through downregulation of KRAS and inactivating AKT and ERK signaling pathways	(89)
*miR-34b*	5 uveal melanoma tissues and ANTs	SP6.5	Yes	c-Met	–	–	Its overexpression expression inhibits melanoma cells proliferation and migration and induces cell cycle arrest by targeting c-Met	(90)
*miR-34c*	5 uveal melanoma tissues and ANTs	SP6.5	Yes	c-Met	–	–	Its overexpression expression inhibits melanoma cells proliferation and migration and induces cell cycle arrest by targeting c-Met.	(90)
*miR-34a*	–	M17, M23, SP6.5, U-96	Yes	LGR4, MMP2	–	–	Its overexpression decreases migration and invasion of uveal melanoma cells through targeting LGR4 and regulation of EMT process.	(91)
*miR-34a*	6 *in situ* melanoma tissues, 6 metastatic melanoma tissues, 6 nevi tissue and 18 ANTs	WM35, WM451, A375	Yes	FLOT2	–	–	Its overexpression represses proliferation and metastasis in melanoma cells *via* targeting FLOT2.	(92)
*miR-34a*	3 uveal melanoma tissues	M17, M21, M23, SP6.5, D78, HEK-293	Yes	c-Met	Akt and ERK1/2 signaling pathways	–	Suppresses proliferation and migration of uveal melanoma cells through downregulation of c-Met	(83)
*miR-34a*	Fifteen patient-derived primary cultures of melanoma, SCID-NOD mice (HAG cell line was injected to mice)	C8161 (HAG), C81-61 (PAG)	Yes	–	–	–	Suppresses proliferation, invasion and tube formation in melanoma cells	(93)
*miR-184*	Fifteen patient-derived primary cultures of melanoma, SCID-NOD mice (HAG cell line was injected to mice)	C8161 (HAG), C81-61 (PAG)	Yes	–	–	–	Suppresses proliferation, invasion and tube formation in melanoma cells	(93)
*miR-182*	Uveal melanoma tissues and normal uveal tissues, Female nude mice (M23 and SP6.5 cell lines were injected to mice)	M23, SP6.5, HEK-293	Yes	MITF, BCL2 and cyclin D2	Akt and ERK1/2 signaling pathways	–	Inhibits cell proliferation, migration and invasion and promotes apoptosis in melanoma cells	(94)
*miR-185*	Fifteen patient-derived primary cultures of melanoma, SCID-NOD mice (HAG cell line was injected to mice)	C8161 (HAG), C81-61 (PAG)	Yes	–	–	–	Suppresses proliferation, invasion and tube formation in melanoma cells	(93)
*miR-185*	52 cutaneous melanoma tissues, 41 uveal melanoma tissues and 35 normal skin specimens	G361, GR-M, OCM-1	Yes	IL-10Rα	–	–	Its ectopic expression decreases proliferation of all melanoma cell lines through targeting IL-10Rα	(95)
*miR-204*	Fifteen patient-derived primary cultures of melanoma, SCID-NOD mice (HAG cell line was injected to mice)	C8161 (HAG), C81-61 (PAG)	Yes	–	–	–	Suppresses proliferation, invasion and tube formation in melanoma cells	(93)
*miR-365*	Skin Cutaneous Melanoma (SKCM) dataset for 470 melanoma samples was downloaded from TCGA	NHEM, A375, A2058, SK-MEL-2, SK-MEL-28	Yes	BCL2, CCND1	–	–	Inhibits cell proliferation, migration and invasion and promotes apoptosis in melanoma cells.	(96)
*miR-365*	40 melanoma tissues and paired ANTs, female BALB/c nude mice (A375 cell line was injected to mice)	A375, G361, LIBR, HME1	Yes	NRP1	–	lymph node metastasis, clinical stage, overall survival, relapse-free survival	Inhibits melanoma growth and metastasis by targeting NRP1	(97)
*miR-485-5p*	20 human primary melanoma tissues and paired ANTs	A375, SK-HEP-1, SK-MEL-1, MV3, HPM	Yes	FZD7	wnt signaling pathway	–	Suppresses proliferation and invasion of melanoma cells through targeting FZD7 and consequently inhibition of wnt signaling	(98)
*miR-612*	89 melanoma tissues and paired ANTs, nude mice (A375 cell line was injected to mice)	SK-MEL-28, SK-MEL-3, A375, HT-144, Hs294T, HEM, HEK293T	Yes	Espin	–	melanoma thickness, lymph node metastasis, poor survival	Its overexpression inhibits melanoma growth, migration and invasion through downregulation of Espin also sensitizes melanoma cells to doxorubicin	(99)
*miR-7-5p*	20 male NOD.CB17-Prkdcscid Il2rgtm1Wjl/SzJ (NSG) mice (1205Lu cell line was injected to mice)	WM266-4, SK-MEL-2, A2058, 1205Lu	Yes	RelA	NF-κB signaling pathway	–	Inhibits cell proliferation, migration and invasion in melanoma *via* inhibiting RelA and reducing activity of NF-κB signaling	(100)
*miR-7-5p*	–	WM266-4, A375, A2058	Yes	IRS-2	Akt signaling pathway	–	Inhibits migration and invasion of melanoma cells through targeting IRS-2 and inhibition of Akt signaling	(101)
*miR-7*	BALB/c nude mice (A375 cell line was injected to mice)	A375, Mel-CV	Yes	EGFR, IGF-1R, CRAF	MAPK and PI3K/AKT signaling pathways	–	Its upregulation reverses BRAF inhibitor resistance in melanoma cells through inhibition of MAPK and PI3K/AKT signaling pathways	(102)
*miR-153-3p*	20 melanoma tissues and matched ANTs	A375, SK-MEL-28, D78	Yes	SNAI1	–	–	Its overexpression represses proliferation and invasion and induces apoptosis by downregulating SNAI1	(103)
*miR-625*	30 melanoma tissues and paired ANTs, SPF grade male BALB/c nude mice (A375 cell line was injected to mice)	A375, M14	Yes	SOX2	–	–	Inhibits proliferation, migration and invasion of melanoma cells by targeting SOX2	(104)
*miR-23a*	30 specific-pathogen‐free (SPF) closed colony male ICR mice (B16 cell line was injected to mice)	B16	Yes	SDCBP	MAPK/ERK Signaling Pathway	–	Its overexpression decreases proliferation, migration and invasion and induces cell cycle arrest at G1 phase and apoptosis in melanoma cells *via* suppression of SDCBP expression and regulation of MAPK/ERK Signaling	(105)
*miR-23a*	Serum samples from 192 melanoma cases and 51 matched cancer-free controls, tissue specimens from 66 melanoma cases and 22 nevus cases, female BALB/C-Nu nude mice (A2058 cell line was injected to mice)	WM35, WM793, 451LU, A2058, A375	Yes	ATG12	AMPK-RhoA pathway	Patient survival, tumor thickness, ulceration, AJCC stage	Decreases migration and invasion in melanoma cells through targeting ATG12 and regulation of autophagy	(106)
*miR-23b*	114 primary melanoma tissues and ANTs, Nude mice (A375 and SK-MEL-28 cell lines were injected to mice)	A375, Hs294t, SK-MEL-5, SK-MEL-28, B16F10, nHEM	Yes	NAMPT	NF-κB signaling pathway	Patient survival, Clark level, sentinel-lymph-node positive, AJCC stage	Suppresses cell proliferation and angiogenesis and promotes apoptosis through targeting NAMPT	(107)
*miR-23a-3p*	117 mucosal melanoma and 12 mucosal nevi, female NOD/SCID mice (HMVII cell line was injected to mice)	GAK, VMRC-MELG, HMVII, HEK293T	Yes	ADCY1	cAMP and MAPK signaling pathways	TNM stage, poor overall survival and disease free survival	Inhibits proliferation, migration and invasion of mucosal melanoma cells through targeting ADCY1 and inhibition of cAMP and MAPK signaling pathways	(108)
*miR-15a*	24 C57BL/6 mice (B16-F10 cell line was injected to mice)	A375, SK-MEL-28, WM1552C, B16-F10	Yes	CDCA4, AKT3	–	–	Inhibits cell proliferation, migration and invasion and contributes to cell cycle arrest at G1/G0 phase through targeting CDCA4	(109)
*miR-15a*	52 cutaneous melanoma tissues, 41 uveal melanoma tissues and 35 normal skin specimens	G361, GR-M, OCM-1	Yes	IL-10Rα	–	–	Its ectopic expression decreases proliferation of all melanoma cell lines through targeting IL-10Rα	(95)
*miR-143-3p*	30 formalin fixed paraffin-embedded (FFPE) primary melanoma lesions and lymph node	NHEM, Sk-Mel-28, A375, WM983A, WM1862	Yes	COX-2	–	–	Represses cell proliferation, migration and invasion and induces apoptosis through targeting COX-2	(110)
*miR-143*	–	NHEM, WM115, SK-Mel-28, A2058	Yes	–	–	–	Its overexpression inhibits cell proliferation and induces apoptosis in melanoma cells	(111)
*miR-708*	60 C57BL/6J male mice (B16 cell line was injected to mice)	B16, B16F10, HEK293	Yes	BAMBI	Wnt Signaling Pathway, TGF-β Signaling Pathway	–	Its overexpression decreases proliferation, migration and invasion and induces apoptosis in melanoma cells through targeting BAMBI and activation of TGF-β Pathway and suppression of Wnt pathway	(112)
*miR-708*	40 clean male Kunming mice (B16 cell line was injected to mice)	B16, A375, WM239, WM451	Yes	LEF1	Wnt signaling pathway	–	Its overexpression expression inhibits proliferation, migration and invasion and induces apoptosis in melanoma cell through targeting LEF1	(113)
*miR-216b*	30 melanoma tissues and ANTs, NOD-SCID mice (A375 cell line was injected to mice)	HEK-293T, A375, A875, SK-MEL-1, HaCaT	Yes	FOXM1	FOXM1 signaling pathway	–	Decreases proliferation, migration and colony formation ability of melanoma cells by targeting FOXM1	(114)
*miR-216a-5p*	86 uveal melanoma tissues, nude mice (A375 cell line was injected to mice)	HEK293T, A375, MUM-2B,	Yes	HK2	–	Patient survival	Suppresses proliferation and dampens glycolysis in melanoma cell *via* targeting HK2	(115)
*miR-150-5p*	nude mice (A375 cell line was injected to mice)	A375, SK-MEL-2, HEK293T	Yes	SIX1	–	–	Inhibits proliferation, migration and invasion of melanoma cells through SIX1-mediated regulation of glycolysis	(116)
*miR-150*	51 melanoma tissues and paired ANTs, BALB/c nude mice (A375 cell line was injected to mice)	MeWo, MHEM, A375, WM-115, WM35, PEM	Yes	MYB	–	Patient prognosis	Represses proliferation, migration and invasion of melanoma cells by inhibition of MYB	(117)
*miR-150-5p*	52 serum samples from stage III and 40 serum samples from stage IV patients, 76 stage III and 10 stage IV FFPE tissue samples	–	No	–	–	Patient survival,	A potential prognostic biomarker	(118)
*miR-142-3p*	52 serum samples from stage III and 40 serum samples from stage IV patients, 76 stage III and 10 stage IV FFPE tissue samples	–	No	–	–	Patient survival, disease stage	A potential prognostic biomarker	(118)
*miR-142-5p*	52 serum samples from stage III and 40 serum samples from stage IV patients, 76 stage III and 10 stage IV FFPE tissue samples	–	No	–	–	Patient survival, disease stage	A potential prognostic biomarker	(118)
*miR-136*	40 male Kunming mice (B16 cell line was injected to mice)	B16, A375, WM239, WM451	Yes	PMEL	Wnt signaling pathway	–	Its overexpression suppresses proliferation, migration, invasion and EMT process and induces apoptosis in melanoma cells by targeting PMEL and inhibition of Wnt signaling pathway	(119)
*miR-214*	RNA‐seq data of 342 melanoma tumors were downloaded from TCGA	MRA2, MRA4, MRA5, MRA6, MRA9	Yes	ANKRD6, CTBP1	–	–	Its overexpression enhances malignant properties of melanoma cells and induces drug resistance in these cells through targeting negative regulators of Wnt signaling.	(120)
*miR-125b*	48 primary melanoma tissues, 36 lymph nodes metastases and 12 neoplastic skin samples, Female athymic BALB/c nude mice (Mel lm cell line was injected to mice)	NHEM, Mel Im, Mel Ju, Mel Ho, A375	Yes	ITGA9	–	–	Inhibits proliferation, invasion and EMT process in melanoma cells by targeting ITGA9	(121)
*miR-125b*	68 primary malignant melanoma tissues and 49 lymph node metastases	NHEM, Mel Im, Mel Ju, Melanoma Ho, A375	Yes	MLK3	c-Jun signaling pathway	–	Its overexpression reduces proliferation and invasion of melanoma cell by targeting MLK3/JNK pathway	(122)
*miR-125b*	5 primary melanoma tissues and 5 melanoma metastases	Mel Juso, Mel Im, Mel Ju, A375, 1205 Lu, HMB2, NHEM	Yes	c-Jun	–	–	Its upregulation reduces proliferation and migration in melanoma cell by targeting c-Jun	(123)
*miR-125b*	–	IGR, SK-Mel28, SK-Mel25, SK-Mel5, MelJuso, SM, MeWo	Yes	VDR	vitamin D signaling	–	Influences VDR expression and resistance of melanoma cell lines to 1,25(OH) (2)D (3)	(124)
*miR-125b*	65 primary melanoma tissues and 67 melanoma metastases	A375, SKMEL-147, 451 Lu	No	–	–	Patient survival, Breslow thickness, ulceration, Mitosis/mm^2^, growth phase,	Can be a potential prognostic biomarker	(125)
*miR-596*	FFPE tissues specimens of 36 melanomas and 22 nevi	A375, SK-Mel-19, A2058, Malme-3M, SK-Mel-12, SK-Mel-2, Malme-3	Yes	MEK1, MCL1, BCL2L1	MAPK/ERK signaling pathway	Poor overall survival	Its overexpression reduces proliferation, migration and invasion and stimulates apoptosis through targeting MEK1, MCL1 and BCL2L1 and regulation of MAPK/ERK and apoptotic pathways	(126)
*miR‐137*	–	A2058, WM793B, HEMa‐LP, HEK‐293T	Yes	FGF9	–	–	Its enforced expression by Propofol decreases proliferation, migration and invasion in melanoma cells through inhibition of FGF9 expression	(127)
*miRNA-29c*	30 malignant melanoma tissues and 10 paracancer tissues	A375, SK-MEL-1, SK-MEL-5, HEMa-LP	Yes	CDK6	–	Poor prognosis, TNM stage	Reduces cell proliferation and induces cell cycle arrest at G1 phase through suppressing expression of CDK6	(128)
*miR-488-5p*	primary melanoma tumors, melanoma metastases, normal skin and nevi	Mel Im, 501mel, NHEM	Yes	DIXDC1	Wnt/β-catenin signaling pathway	–	Has a tumor suppressive role. Its overexpression represses proliferation and migration and induces apoptosis in melanoma cells	(129)
*miR-488-3p*	20 malignant melanoma tissues and ANTs, 12 male Nu/Nu mice	A375, B16, SK-MEL-28, WM451, HEMn-LP	Yes	PRKDC	–	–	Its ectopic expression sensitizes melanoma cells to cisplatin *via* targeting PRKDC	(130)
*miR-675*	21 melanoma tissues and ANTs	A375, A2058, HT144, SK-MEL-28, HEM	Yes	MTDH	–	–	Has a tumor suppressive role. Its overexpression inhibits proliferation and invasion in melanoma cells partly by targeting MTDH	(131)
*miR-622*	Primary tumor and metastatic tumor tissue, male athymic nu/nu mice (Mel Im cell line was injected to mice)	Mel Juso, Mel Ei, Htz19, Mel Im, NHEM	Yes	KRAS	–	Patient survival	Its re-expression suppresses proliferation, migration and clonogenicity in melanoma cells.	(132)
*miR-92*	Female C57Bl/6 mice (B16-F10 cell line was injected to mice)	B16-F10	Yes	integrin α_V_ and α_5_	TGFβ signaling pathway	–	Implicates in integrin activation of TGFβ in melanoma cancer stem cells that gives rise to immunosuppressive tumor microenvironment and increased tumorigenesis	(133)
*miR-4487*	86 melanoma samples, serum samples from 130 normal controls and 255 melanoma cases	–	No	–	–	Diseases stage, survival	A probable diagnostic biomarker	(23)
*miR-4706*		–	No	–	–	Diseases stage, survival		(23)
*miR-4731*		–	No	–	–	Diseases stage		(23)
*miR-509-3p*		–	No	–	–	Diseases stage		(23)
*miR-509-5p*		–	No	–	–	Diseases stage		(23)
*miR-203*	–	KMeC, LMeC, CMeC-1, A2058, Mewo, HEM	Yes	CREB	–	–	Inhibits melanoma growth and melanosome transport regulating CREB/MITF/RAB27a pathway	(134)
*miR-203*	8 primary melanoma tissues, 11 metastases and 5 normal skin tissues	A375, A2058, SKMEL13, HT144, SKMEL5	Yes	BMI1	–	Tumor metastasis	Represses melanoma cells invasion and tumor sphere formation by targeting BMI1	(135)
*miR-203*	148 melanoma tissues and paired ANTs	–	No	–	–	Overall survival, tumor thickness, tumor stage	Can be a potential prognostic factor and a new therapeutic target for the treatment of melanoma	(136)
*miR-203*	–	A2058, Mewo, HEMa-LP	Yes	kif5b	–	–	Its exogenous expression suppresses melanoma cells growth and regulates melanosomes transport and tyrosinase expression through targeting kif5b	(137)
*miR-203*	–	Mewo, A2058, HEM	Yes	E2F3a, E2F3b, ZBP-89	–	–	Induces cell cycle arrest and senescence in melanoma cells *via* targeting E2F3	(138)
*miR-203*	24 melanoma tissues and paired ANTs	A375, HaCaT,	Yes	versican	–	–	Inhibits migration of melanoma cells through targeting versican	(139)
*miR-17-3p*	28 uveal melanoma tissues and 12 control samples, 30 male BALB/c nude mice (OCM-1A cell line was injected to mice)	OCM-1A, MUM-2C, C918, MUM-2B, UMs	Yes	PVT1, MDM2	p53 signaling pathway	–	Its overexpression inhibits proliferation, migration and invasion and promotes apoptosis through PVT1/miR-17-3p/MDM2 axis	(140)
*miR-137*	30 primary melanoma tissues and paired ANTs	A375, SK-MEL-1, SK-MEL-5, HEMa-LP, HEMn-LP	Yes	GLS	–	Poor survival, TMN stage	Has a tumor suppressive role. It inhibits proliferation and glutamine catabolism in melanoma cells *via* targeting glutaminase	(141)
*miR-137*	–	M17, M23, SP6.5, um95, HEK-293	Yes	MITF, CDK6	–	–	Its ectopic expression inhibits uveal melanoma cells proliferation and induces cell cycle arrest at G1 phase through downregulation of MITF and CDK6	(142)
*miR-137*	–	Ma-Mel-79b, Ma-Mel-86b	Yes	GLO1	–	–	Its overexpression suppresses proliferation of melanoma cells by targeting GLO1	(143)
*miR-137*	*miR-137* expression data of 450 melanoma patients was obtained from TCGA	WM1650, ME1402, MM200, WM1158	Yes	TBX3	–	Patient survival	Inhibits melanoma cell migration and anchorage independent growth by targeting TBX3	(144)
*miR-137*	30 melanoma tissues and 10 normal skin tissues, BALB/c female mice (A375 cell line was injected to mice)	A2058, A375, A875, SKMEL5, TE353-SK, Hacat	Yes	AURKA	–	–	Decreases proliferation and colony formation ability of melanoma cells through targeting AURKA	(145)
*miR-137*	97 melanoma tissues and paired ANTs	–	No	–	–	Patient survival, TNM stage, ulcer, occurrence site	Its low expression is associated with poor prognosis in melanoma patients.	(146)
*miR-137*	–	Ma-Mel-12, MaMel-20, Ma-Mel-37b, Ma-Mel-57, Ma-Mel-73a, Ma-Mel-79b, MaMel-86b, SK-Mel-2, SK-Mel5	Yes	PAK2	–	–	Suppresses proliferation of melanoma cells *via* inhibiting PAK2	(147)
*miR-137*	–	WM278, A375, HEK293	Yes	CtBP1	–	–	Suppresses EMT process and induces apoptosis in melanoma cells through targeting CtBP1	(148)
*miR-137*	–	melanoma cell lines established from metastasis of 33 patients with stage III or IV melanoma	Yes	c-Met, YB1, EZH2, MITF	–	Patient survival	Inhibits cell proliferation, migration and invasion and induces apoptosis in melanoma cells *via* targeting c-Met, YB1, EZH2 and MITF	(149)
*miR-137*	15 melanoma tissues and 15 normal pigmented nevus samples	HaCaT, SK‐MEL‐1, A375, WM451	Yes	PIK3R3	–	–	Represses migration and invasion of melanoma cells *via* targeting PIK3R3	(150)
*miR−30a−5p*	22 malignant melanoma tissues and ANTs, BALB/c nude mice (A375 cell line was injected to mice)	A375, SK-HEP-1, SK-MEL-1, MV3, HPM	Yes	SOX4	–	–	Inhibits melanoma cells proliferation, migration and invasion *via* targeting SOX4	(151)
*miR-218*	10 primary melanoma tissues, 10 lymph node metastases and 10 benign nevi samples	A375, SK-MEL-2	Yes	CIP2A, BMI1			Inhibits proliferation, migration and invasion in melanoma cells by targeting CIP2A, BMI1	(152)
*miR-605*	male BALB/c nude mice (Mel-RM and SK-Mel-28 were injected to mice)	HEMn-MP, SK-MEL-31, ME4405, WM1321, Me1007, Mel-RM, SK-MEL-2, SK-MEL-103, WM1366, Mel-RMU, WM278, A375, MM200, SK-Mel-28	Yes	INPP4B	–	–	Inhibits proliferation and growth of melanoma cells through suppression of INPP4B and consequently INPP4B-mediated negative regulation of SGK3	(153)
*miR-24−1−5p*	77 malignant melanoma tissues and paired ANTs	A375	Yes	UBD	JNK signaling pathway		Its overexpression gives rise to promotion of autophagy and apoptosis in melanoma cells *via* targeting UBD and activation of JNK signaling pathway	(154)
*miR-205-5p*	6 melanoma tissues and 6 skin nevus samples	HaCaT, A431, A375, A2058 and SK-MEL-2	Yes	TNFAIP8	–	–	Enhances apoptosis rate and sensitizes melanoma cells to vemurafenib through targeting TNFAIP8	(155)
*miR-205-5p*	32 primary cutaneous melanoma tissues and 8 metastatic samples	–	No	–	–	Distant metastasis	Can be a potential biomarker of distant metastases	(156)
*miR-145-5p* *miR-203-3p*	32 primary cutaneous melanoma tissues and 8 metastatic samples	–	No	–	–	Breslow thickness, high Clark level, ulceration, mitotic rate	Can be potential markers of aggressiveness in melanoma	(156)
*miR-205*	10 primary melanoma tissues, 10 metastatic melanoma tissues and 10 benign nevi samples, 16 male athymic nu/nu mice (WM115A cell line was injected to mice)	WM35, WM793, WM115A, 1205Lu, 293T	Yes	–	–	–	Its enforced expression reduces migration, motility and proliferation of melanoma cells	(157)
*miR-205*	20 primary melanoma tissues, 27 metastatic melanoma tissues and 20 benign nevi	WM3211, DO4, WM278, 1205-Lu, C8161.9, Normal human melanocytes	Yes	E2F1, E2F5	AKT signaling pathway	–	Its overexpression suppresses proliferation and colony formation and induces apoptosis in melanoma cell *via* targeting E2F1	(14)
*miR-205*	65 primary melanoma tissues and 67 melanoma metastases	A375, SKMEL-147, 451 Lu	No	ZEB1	–	Patient survival, Breslow thickness, ulceration, Mitosis/mm^2,^ growth phase, Histological type	Influences invasive ability of melanoma cells and can be a potential prognostic biomarker	(125)
*miR-205*	5 high-invasive uveal melanoma tissues, 5 low-invasive uveal melanoma tissues and 5 healthy controls	OCM-1A, C918, 293T	Yes	NRP1	–	–	Its overexpression represses proliferation and invasion of melanoma cells *via* targeting NRP1	(158)
*miR-145-5p*	83 melanoma samples and paired ANTs, 30 male BALB/c nude mice (CHL‐1, WMM917, or SK‐mel‐28 cell lines were injected to mice)	HEK293T, SK‐mel‐28, CHL‐1, VMM917, NHEM	Yes	NRAS	MAPK and PI3K/AKT signaling pathways	Tumor thickness, NRAS mutation, tumor stage	Its high expression inhibit proliferation, migration and invasion and promotes apoptosis in VMM917 and CHL-1 melanoma cells through targeting NRAS	(159)
*miR-145-5p*	55 melanoma samples and paired ANTs, 10 female athymic BALB/c nude mice (A375 cell line was injected to mice)	A375, WM35, VMM5A, M14, A875, HMCB, 293T	Yes	TLR4	NF-κB signaling pathway	–	Suppresses proliferation, migration and invasion of melanoma cell *via* targeting TLR4	(160)
*miR-145-5p*	12 uveal melanoma tissues and 12 normal uveal tissues	OCM-1, MUM- 2B	Yes	N-RAS, VEGF	–	–	Inhibits tumor growth, angiogenesis and invasion of uveal melanoma cells through targeting N-RAS and VEGF	(161)
*miR-195*	341 matched mRNA-Seq and miRNA-Seq tumor samples, along with one normal sample for each data set were obtained from TCGA	SK-MEL-5, SK-MEL-19, SK-MEL-37, SK-MEL-147, UACC-62, WM35, WM793B, WM1366, WM1552C, WM1617, Lox10, MZ2Mel, HaCat, NGM	Yes	PHB1	–	–	Its upregulation results in decreased cell proliferation and high cytotoxic effects of cisplatin and temozolomide on melanoma cells	(162)
*miR-211*	Male BALB/c nude mice (SK-MEL-28 cell line was injected)	A375, SK-MEL-28	Yes	–	–	Poor prognosis, tumor thickness, AJCC stage	Its upregulation sensitizes melanoma cells to cisplatin and increases cisplatin anticancer effect	(163)
*miR-211*	–	A375, WM1552C, HEM-l	Yes	PDK4	–	–	Acts as a metabolic switch and sensitizes melanoma cells to hypoxia through targeting PDK4	(164)
*miR-211*	6 primary melanoma tissues and 24 melanoma metastases	HEM-l, A375, G361, LOX-IMV1, HT-144, RPMI-7951, SK-MEL2, SK-MEL28, WM793B, WM1552C,	Yes	KCNMA1	–	–	Its overexpression reduces growth and invasion of melanoma cells *via* targeting KCNMA1	(165)
*miR-211*	–	HM, WM115, A375, SK-MEL-1	Yes	RAB22A	–	–	Regulates EMT process through targeting RAB22A	(166)
*miR-211*	–	HMV-I, HMV-II, G-361, SK-MEL-28, NHEM-L, NHEM-M, NHEM-D, MM-EP, MM-RU, MM-WK, HEK-293	Yes	PRAME	–	–	Regulates PRAME expression in melanoma cells its overexpression cause reduction in PRAME expression	(167)
*miR-211*	–	61 melanoma cell lines (some of them include: A2–A15, D4–D25, ME1007, ME1402, ME4405, ME10538, Mel-FH, Mel-RM, Mel-RMU, MM470, MM537, MM629)	Yes	BRN2	–	–	Changes invasion capacity of melanoma cells through targeting BRN2	(168)
*miR-211*	miRNA expression was derived for eleven melanoma cell lines and matched to samples obtained from GEO	WM3526, WM3682, 451LU	Yes	NUAK1	–	–	Its upregulation inhibits invasion and restores adhesion through targeting NUAK1	(169)
*miR-211*	52 cutaneous melanoma tissues, 41 uveal melanoma tissues and 35 normal skin specimens	G361, GR-M, OCM-1	Yes	IL-10Rα	–	–	Its ectopic expression decreases proliferation of all melanoma cell lines through targeting IL-10Rα	(95)
*miR-211*	–	A375M, UACC62, HeLa	Yes	IGF2R, TGFBR2, NFAT5	–	–	Its overexpression inhibits migration and invasion of invasive melanoma cells	(170)
*miR-181a*	10 melanoma tissues and paired ANTs	WM266-4, A2058	Yes	Bcl-2	–	–	Is upregulated by Piceatannol treatment and contributes to anticancer role of piceatannol through targeting Bcl-2	(171)
*miR-181*	17 matched melanoma tissues before and after resistance of patients to BRAF inhibitors	A375, M14	Yes	TFAM	–	Patient survival	Its overexpression impedes melanoma growth and alleviates resistance to dabrafenib through targeting TFAM	(172)
*miR-375*	24 melanoma tissues, normal skin and nevi samples	HEM‐l, HEK, WM793B, WM278, WM1552C	Yes	–	–	–	Its ectopic expression suppresses proliferation invasion, and cell motility and induces changes in cell shape in melanoma cells	(173)
*miR-328*	–	HEM, SK-MEL-1, A375	Yes	TGFB2	–	–	Its overexpression represses proliferation and induces cell cycle arrest at G1 phase	(174)
*miR-4633-5p*	56 Primary human sinonasal mucosal melanoma tissues	A375, M435S	Yes	–	Akt pathway	Metastasis	Inhibits cell growth, invasion and secretion of MMP2 in melanoma	(175)
*miR-455*	20 melanoma tissues and paired ANTs	SKMEL1, A375, HT144, A2058, HEK293T	Yes	IGF−1R	–	–	Suppresses proliferation and invasion in melanoma cells *via* targeting IGF1R	(176)
*miR-145*	5 high-invasive uveal melanoma tissues, 5 low-invasive uveal melanoma tissues and 5 healthy controls	OCM-1A, C918, 293T	Yes	NRP1	–	–	Represses proliferation and invasion of melanoma cells *via* targeting NRP1	(158)
*miR-145*	33 oral canine malignant melanoma tissues and 11 canine normal oral mucosa tissues,	KMeC, LMeC, CMeC-1, CMeC-2, A2058, Mewo, HEM	Yes	c-MYC, FASCIN1	–	–	Inhibits proliferation and migration in melanoma cells through suppression of c-MYC and FASCIN1	(177)
*miR-145*	11 uveal melanoma tissues and 12 normal controls	MUM-2B, OCM-1	Yes	IRS-1	–	–	Inhibits cell proliferation through blocking G1/S transition and induces apoptosis in uveal melanoma cells *via* targeting IRS-1	(178)
*miR-145*	–	BLM, FM3P, WM793	Yes	–	–	–	Its overexpression inhibits migration and invasion in metastatic melanoma cells	(179)
*miR-219-5p*	42 melanoma tissues and 20 nevi tissues, 6 nude mice (A375 cell line was injected to mice)	A375, WM35, SK-MEL-5, SK-MEL-2, HEMa-LP	Yes	Bcl-2		Overall survival, TNM stage, distant metastasis	Reduces proliferation, migration and invasion and promotes apoptosis in melanoma cells by targeting Bcl-2	(180)
*miR-31*	9 primary melanoma tissues, 71 metastatic melanoma and 2 dysplastic nevi	SK-Mel5, SK-Mel28, MM603	Yes	SRC, MET, NIK, RAB27a	–	–	Has tumor suppressive role and its ectopic expression inhibits migration and invasion in melanoma cells	(181)
*miR-31*	Fifteen patient-derived primary cultures of melanoma, SCID-NOD mice (HAG cell line was injected to mice)	C8161 (HAG), C81-61 (PAG)	Yes	–	–	–	Suppresses proliferation, invasion and tube formation in melanoma cells	(93)
*miR-124a*	6 primary uveal melanoma and paired ANTs, Female nude mice (M23 and SP6.5 cell lines were injected to mice)	M17, M21, M23, SP6.5, HEK-293, um95	Yes	CDK4, CDK6, cyclin D2, EZH2	–	–	Has a tumor suppressive role and represses proliferation, migration and invasion in uveal melanoma cells	(182)
*miR-124*	107 melanoma tissues and paired ANTs	HEM, SK-MEL-1, A375	Yes	RLIP76	–	TNM stage	Suppresses melanoma cells proliferation and invasion and induces apoptosis by targeting RLIP76	(183)
*miR-124*	68 melanoma tissues and paired ANTs	B16, A375, HACAT	Yes	Versican	–	Tumor thickness, clinical stage, lymph node involvement	Inhibits proliferation, migration and invasion of melanoma cells through targeting Versican	(184)
*miR-206*	serum samples from 60 melanoma patients and 30 healthy controls	–	No	–	–	Poor prognosis, response to treatment, clinical stage	May be implicated in melanoma progression an can be a potential prognostic factor	(185)
*miR-206*	36 melanoma tissues and 16 Healthy control tissues	A375, MALME-3M, RPMI7951, SKMEL-2, SK-MEL-5, NHEM-Ad-Adult	Yes	CDK4, Cyclin D1, Cyclin C	–	–	Reduces proliferation, migration and invasion and induces cell cycle arrest at G1 phase in melanoma cells	(186)
*miR-186*	–	SK-MEL-1, G-361, A375, A875, HEMn-LP	Yes	–	–	–	Inhibits proliferation, migration and invasion in melanoma cells	(187)
*miR-26b*	59 melanoma tissues and ANTs	HEK293, B16F10, B16F0, A375, HMCB, Hs695T	Yes	TRAF5	MAPK pathway	–	Reduces cell growth and induces apoptosis in melanoma cells by targeting TRAF5	(188)
*miR-196a*	3 primary melanoma tissues and 5 melanoma metastases	Mel Ei, Mel Wei, Mel Juso, Mel Im, Mel Ju, HMB2, SkMel 3, SkMel 28, NHEM	Yes	HOX-C8	–	–	Decreases invasion in melanoma cells through suppression of HOX-C8 expression	(189)
*miR-196a*	–	Mel Ei, Mel Wei, Mel Ho, Mel Juso, Mel Ju, SkMel 28, SkMel 3, NHEM	Yes	HOX-B7	–	–	Regulates migration of melanoma cells through influencing miR-196a/HOX-B7/Ets-1/bFGF/BMP4 axis	(190)
*miR-193b*	FFPE specimens of 8 benign nevi, and 8 metastatic melanomas	Malme-3M, SKMEL-28, SKMEL-5	Yes	CCND1	–	–	Suppresses proliferation and cell cycle arrest at G1 phase in melanoma cells through downregulation of CCND1	(191)
*miR-193b*	FFPE tissue specimens of 8 benign nevi, 8 metastatic melanoma and 15 primary melanoma tissues	Malme-3M, MeWo, SK-MEL-2, SK-MEL-28	Yes	Mcl-1	–	–	Sensitizes melanoma cells to ABT-737 and regulates expression of Mcl-1	(192)
*miR-200c*	10 primary melanoma tissues, 10 metastatic melanomas and 10 benign nevi samples, male athymic nu/nu mice (WM115A cell line was injected to mice)	WM35, WM793, WM115A, WM3523A, 1205Lu, 293T	Yes	BMI-1	–	–	Inhibits proliferation, migration and metastasis of melanoma cells *via* suppression of BMI-1 expression	(193)
*miR-200c*	65 primary melanoma tissues and 67 melanoma metastases	A375, SKMEL-147, 451 Lu	No	–	–	Patient survival, Breslow thickness, ulceration, Mitosis/mm^2,^ growth phase, location, histological type	Can be potential prognostic biomarker	(125)
*miR-200a*	Paraffin-embedded archival tissue specimens of 7 primary melanoma, 33 lymph node metastasis, 25 distant organ metastasis and 10 benign nevi	MELANO, MEL 2183, COLO 829	Yes	CDK6	–	–	Inhibits proliferation and induces cell cycle arrest in melanoma cells through targeting CDK6	(194)
*miR-200a*	46 melanoma tissues and paired ANTs	A375, SK-HEP-1, WM35, SK-MEL-28	Yes	GOLM1	PI3K/Akt signaling pathway	Overall survival, tumor thickness, TNM stage	Inhibits proliferation, migration and invasion of melanoma cells *via* targeting GOLM1 and regulation of PI3K/Akt signaling pathway	(195)
*miR-155*	25 uveal melanoma tissues and ANTs	OCM-1A, MUM-2C, C918, MUM-2B, D78	Yes	NDFIP1	–	–	Enhances proliferation and invasion of uveal melanoma cells *via* targeting	(196)
*miR-155*	–	SK-Mel-28, WM-266-4, GL-Me, 397-Mel, CH-Mel, DR-Mel, SN-Mel,	Yes	SKI	–	–	Inhibits melanoma cells proliferation through targeting SKI	(197)
*miR-155*	–	CG-Mel, CH-Mel, CL-Mel, CN-Mel, CR-Mel, CT-Mel, DR-Mel, GL-Mel, GR-Mel, MR-Mel, M14, PNM-Mel, PNP-Mel, SK-Mel-28, SN-Mel, WM-266-4, 397-Mel, normal melanocytes	Yes	–	–	–	Its ectopic expression represses proliferation and induces apoptosis in melanoma cells	(16)
*miR-155*	60 melanoma tissues and paired ANTs	A375, SKMEL-28, A2058, HEM	Yes	CBL	–	tumor thickness, TNM stage, lymph node metastasis	Suppresses proliferation, migration and invasion of melanoma cells *via* targeting CBL	(198)
*miR-18b*	92 primary melanoma tissues and 48 benign nevi samples, 20 nude mice (1205-Lu cell line was injected to mice)	1205-Lu, DO4, WM3211, WM278	Yes	MDM2	p53 signaling pathway	Overall survival	Reduces proliferation, migration and invasion and induces apoptosis in melanoma cell through downregulation of MDM2	(199)
*miR-18b*	68 melanoma tissues and paired ANTs, 6 male BALB/C-nu/nu nude mice (B16 cell line was injected to mice)	HEK293 cells, MM B16, A375, HACAT	Yes	HIF-1α	–	tumor thickness, tumor stage	Inhibits glycolysis and cell proliferation and induces cell cycles arrest in melanoma cells through targeting HIF-1α	(200)
*miR-26a*	–	SK-MEL-28, HT-144, HEK293, HEMNLP, HEMNLP2, WM278, WM852c, 1205Lu, A375, RPMI7951	Yes	SODD	–	–	Decreases cell viability and induces apoptosis in melanoma cells through targeting SODD.	(201)
*miR-26a*	–	A2058, A375, SK-MEL-5, SK-MEL-28	Yes	Lin28B, Zcchc11	–	–	Increases microRNA synthesis by targeting Lin28B and Zcchc11 to inhibit tumor growth and metastasis	(202)
*miR-26a*	male C57BL/6 mice (B16-F10 cell line was injected to mice)	WM1552C, SKMEL-28, B16-F10	Yes	MITF	–	–	Inhibits proliferation and invasion of melanoma cells *via* targeting MITF	(203)
*miR-9*	10 primary melanoma tissues and 10 metastases	WM35, WM793, WM115A, 1205Lu, 293T	Yes	NF-κB1	NF-κB1-Snail1 signaling pathway	–	Reduces proliferation and migration of melanoma cells through regulation of NF-κB1-Snail1 pathway.	(204)
*miR-9*	24 melanoma tissues and 14 benign nevi samples	WM852, WM1791C, WM8, FO-1, WM983A, WM793, Daju, WM209	Yes	RYBP	–	–	Suppresses proliferation migration and invasion in melanoma cells through targeting RYBP	(205)
*miR-9*	–	MUM-2B, C918, MUM-2C, OCM-1A	Yes	NF-κB1	NF-κB1 signaling pathway	–	Suppresses migration and invasion of uveal melanoma cells *via* targeting cells NF-κB1 and downregulation of the NF-κB1 signaling pathway	(206)
*miR-9*	73 melanoma tissues and paired ANTs, Male BALB/C-nu/nu nude mice (A375 cell line was injected to mice)	HACAT, G361, B16, A375, HME1	Yes	NRP1	–	tumor stage, lymph node metastasis	Decreases melanoma cells proliferation, migration and invasion of through targeting NRP1.	(207)
*miR-9*	24 primary melanoma tissues and paired ANTs	B16, A375, G361, HME1, HACAT, HEK293	Yes	SIRT1	–	–	Inhibits proliferation and migration of melanoma cells partly through targeting SIRT1	(208)
*let-7b*	10 primary melanoma tissues and 10 benign melanocytic nevi	SK-Mel-147, G361	Yes	cyclin D1, cyclin D3, cyclin A, Cdk-4	–	–	Suppresses progression of cell cycle and anchorage-independent growth in melanoma	(85)
*let-7b*	16 melanoma tissues and 8 normal tissues	s SK-mel-28, A375, A2058, HaCaT	Yes	UHRF1	–	–	Suppresses proliferation of melanoma cell by targeting UHRF1	(209)
*let-7b*	–	OCM1, OM431	Yes	cyclin D1	–	–	Sensitizes radioresistance uveal melanoma cells to radiotherapy by targeting cyclin D1	(210)
*let-7b*	106 mucosal melanoma tissues, mucosal nevi Female NOD/SCID (HMVII cell line was injected to mice)	HMVII, GAK, 293T	Yes	MTDH, CALU	–	Patient survival, ECOG score	Suppresses melanoma cells proliferation, migration, invasion and induces apoptosis through targeting MTDH and CALU	(211)
*let-7c*	106 mucosal melanoma tissues, mucosal nevi Female NOD/SCID (HMVII cell line was injected to mice)	HMVII, GAK, 293T	Yes	MTDH, CALU	–	Patient survival, ECOG score	Suppresses melanoma cells proliferation, migration, invasion and induces apoptosis through targeting MTDH and CALU	(211)
*let-7a*	–	Mel Im, Mel Wei, Mel Juso, Mel Ei, Mel Ho, Mel Ju, HMB2, SK-Mel 28	Yes	integrin β3	–	–	Its overexpression decreases invasive ability of melanoma cells through downregulation of integrin β3	(86)
*miR-330-3p*	77 melanoma tissues and 38 Normal skin tissues,	SK-MEL-2, UACC903	Yes	TPX2	–	–	Suppresses proliferation of melanoma cells through negative regulation of TPX2	(212)
*miR-330-5p*	26 primary melanoma tissues and 26 matched non-tumor tissues	HEMn-LP, A375, A875	Yes	TYR, PDIA3	–	–	Represses proliferation, migration and invasion of melanoma cells *via* suppression of TYR and PDIA3 expression	(213)
*miR-183*	30 melanoma tissues and 14 normal skin samples, female BALB/c mice (SK-MEL-1 cell line was injected to mice)	A375, C32, EDMEL3, G361, HBL, WM1115, SK-MEL-1, M14, MV3, A875, M21, Hermes1, Hermes4, Hacat, TE353.SK, HEK293T	Yes	ITGB1	ITGB1 signaling pathway	Poor prognosis, advanced pathological stage	Suppresses proliferation of melanoma cells through targeting ITGB1	(214)
*miR-144*	5 uveal melanoma tissues and 5 uveal normal tissues	MUM-2B, C918, MUM-2C, OCM-1A, D78	Yes	c-Met	–	–	Inhibit proliferation and migration in uveal melanoma cells *via* targeting c-Met	(215)
*miR-144*	26 uveal melanoma tissues and normal choroid samples	MEL270, OMM2.5, UPMM3, UPMM2	Yes	ADAM10, c-Met	–	–	Suppresses proliferation, migration and cell cycle progression in melanoma cells through targeting ADAM10 and c-Met	(216)
*miR-122*	26 uveal melanoma tissues and normal choroid samples	MEL270, OMM2.5, UPMM3, UPMM2	Yes	ADAM10, c-Met	–	–	Suppresses proliferation, migration and cell cycle progression in melanoma cells through targeting ADAM10 and c-Met	(216)
*miR-107*	15 primary melanoma tissues, 15 melanoma metastases and 15 nevi samples	SK-MEL-1, A375, G-361, SK0MEL-3, SH-4, SK-MEL-24	Yes	POU3F2	–	late stage	Decreases proliferation, migration and invasion in melanoma cells	(217)
*miR-296-3p*	18 choroidal malignant melanoma tissues and 6 normal choroidal tissues	C918,	Yes	MMP−2, MMP−9	–	–	Suppresses proliferation, migration and invasion and stimulates apoptosis	(218)
*miR-542-3p*	24 melanoma tissues and 12 non-neoplastic skin tissues, C57BL/6J mice (B16F10 cell line was injected to mice)	A375, SK-MEL-19, SK-MEL-28, WM451, B16F10	Yes	PIM1	–	–	Inhibits migration, invasion and EMT process in melanoma cells *via* targeting PIM1	(219)
*miR-625‐5p*	Primary melanoma tissues and normal tissues	A2085, A375, A875, Mel‐RM, M14, M21, WM35, HFE	Yes	PKM2	–	TNM stage, tumor size, poor differentiation	Inhibits melanoma cells proliferation and glycolysis and sensitizes these cell to BRAF inhibitor *via* targeting PKM2	(220)
*miR-339-3p*	NSG mice (A375 cell line was injected to mice)	A375, WM266.4, WM115	Yes	MCL1	–	–	Reduces invasive ability and metastasis in melanoma cells through targeting MCL1	(221)
*miR-590-5p*	female athymic Balb/C nude mice (A2058 cell line was injected to mice)	A2058, A375, HEMa-LP, 293, HM	Yes	YAP1	–	–	Reduces proliferation and induces apoptosis in melanoma cells *via* downregulation of YAP1	(222)
*miR-768-3p*	–	MM200, Mel-CV, IgR3, Mel-RMu, Sk-Mel-28, Me1007, Mel-JD, Mel-FH, Me4405, Mel-RM, HEMn-MP, HEMn-DP	Yes	eIF4E	–	–	Suppresses cell proliferation and survival and reduces nascent protein synthesis in melanoma cells through targeting eIF4E	(223)
*miR-451a.1*	105 melanoma tissues and 101 normal skin tissues	A2058, A375P, C32, A375SM, WM983A, WM278, WM35, WM1552C	Yes	CAB39	–	–	Inhibits migration and invasion of melanoma cells (this effect is not mediated by CAB39)	(224)
*miR-32*	Genetically engineered mice (Mice carrying an HGF/SF transgene) (A375P cell line was injected to mice)	WM3928, A375P, YUGEN8	Yes	MCL-1	MAPK pathway	–	Decreases tumorigenicity and induces apoptosis in melanoma cells *via* targeting MCL-1	(225)
*miR-493*	52 melanoma tissues and paired ANTs	PEM, SK-MEL-28, WM-115, UACC257, A375, A7, MeWo, NHEM	Yes	IRS4	–	–	Suppresses proliferation and cell cycle progression in melanoma cells through targeting IRS4	(226)
*miR-382*	211 primary melanoma tissues, NOD/Shi-scid/IL-2Rγ^null^ (NOG, Taconic) mice (451Lu cell line was injected to mice)	501MEL, 451Lu, WM1361a, SK-MEL-147, SK-MEL-173, SK-MEL-28	Yes	CTTN, RAC1, ARPC2	–	Tumor thickness, recurrence-free survival	Inhibits tumor metastasis, invasion and matrix degradation through targeting CTTN, RAC1 and ARPC2	(227)
*miR-516b*	211 primary melanoma tissues, NOD/Shi-scid/IL-2Rγ^null^ (NOG, Taconic) mice (451Lu cell line was injected to mice)	501MEL, 451Lu, WM1361a, SK-MEL-147, SK-MEL-173, SK-MEL-28	Yes	–	–	Tumor thickness	Suppresses tumor growth and metastasis	(227)
*miR-194*	60 melanoma tissues and paired ANTs	A375, A875	Yes	GEF-H1	–	TNM stages	Inhibits proliferation and metastasis of melanoma cells through suppression of GEF-H1/RhoA pathway	(228)
*miR-194*	24 melanoma tissues and paired ANTs,	SK-Mel2,	Yes	–	PI3K/AKT/FoxO3a and p53/p21 signaling pathways	Patient survival	Inhibits cell proliferation and induces apoptosis through regulation of PI3K/AKT/FoxO3a and p53/p21 signaling pathways	(229)
*miR-128*	14 primary cutaneous melanoma tissues and ANTs	A375,	Yes	CCL18	–	–	Inhibits migration and colony formation ability and promotes apoptosis in melanoma cells through targeting CL18	(230)
*miR-1280*	37 melanoma tissues and 24 benign nevi samples	A375, Mamel66a, Mamel103b, 1205-Lu, C8161.9	Yes	Src	–	–	Suppresses proliferation, cell cycle progression and invasion and promotes apoptosis in melanoma cells *via* targeting Src	(231)
*miR-573*	11 melanoma tissues and paired ANTs, BABL/c nude mice (A375, SK-MEL-2 cell lines were injected to mice)	A375, SK-MEL-2	Yes	MCAM	–	–	Suppresses proliferation and invasion of melanoma cells *via* targeting MCAM	(232)
*miR-33a-5p*	29 melanoma tissues and ANTs, nude mice (SKMEL-28 cell line was injected to mice)	SKMEL-28, A375, WM35, SKMEL-1, PIG1	Yes	SNAI2	PI3K/AKT/mTOR signaling pathway	Lymph node metastasis, tumor size, STM stage	Suppresses proliferation, migration and invasion and induces apoptosis in melanoma cell through targeting SNAI2	(233)
*miR-33a-5p*	20 melanoma and match nevus tissues	A375, WM35, WM451, SK-MEL-1, HM	Yes	–	–	–	Reduces proliferation and promotes radiosensitivity by suppressing glycolysis in melanoma cells	(234)
*miR-33a*	Male BALB/C-nu/nu mice (A375 cell line was injected to mice)	WM35, WM451, A375, SK-MEL-1, HM	Yes	HIF-1α	–	–	Inhibits proliferation, invasion and metastasis in melanoma cells *via* targeting HIF-1α	(235)
*miR-33a*	–	SK-MEL-1, WM-115, PEMI, PEM2	Yes	PCTAIRE1	–	–	Suppresses proliferation and colony formation ability of melanoma cells through targeting PCTAIRE1	(236)
*miR-33b*	–	WM35, WM451, SK-MEL-1, HM, HEK293	Yes	HIF-1α	–	–	Suppresses melanoma cells proliferation and glycolysis through targeting HIF-1α	(237)
*miR-98*	20 melanoma tissues and 20 normal nevi, 80 male mice (B16-F1 cell line was injected to mice)	B16-F1	Yes	IL-6	–	Patient survival, tumor stage	Suppresses migration and metastasis of melanoma cell through miR-98-IL-6-negative feedback loop	(238)
*miR-425*	Melanoma tissues and normal tissues	A375, SK-MEL-28, UACC257, WM-115, NHEM	Yes	IGF-1	PI3K-Akt signaling pathway	–	Suppresses proliferation and metastasis of melanoma cell *via* targeting IGF-1 and inhibition of PI3K-Akt signaling pathway	(239)
*miR-337*	40 melanoma tissues and paired ANTs	HEK293, A375, A875	Yes	STAT3	–	Patient prognosis	Suppresses growth and metastasis in melanoma cells *via* targeting STAT3	(240)
*miR-637*	61 melanoma tissues and ANTs	A375, SK-MEL-28, Mel-RM, HaCaT	Yes	P-REX2a	PTEN/AKT signaling pathway	lymph node metastasis, TNM stage	Inhibits proliferation and G1-S transition in melanoma cells through targeting P-REX2a	(241)
*miR-329*	36 paraffin‐embedded melanoma tissues and 10 pigmented nevi samples	PEM, WM‐115, A375, A7, UACC257	Yes	HMGB2	β‐catenin signaling pathway	–	Suppresses proliferation, migration and invasion and promotes apoptosis in melanoma cell through negative regulation of HMGB2	(242)
*miR-579-3p*	FFPE samples from 9 stage III/IV melanomas, 10 stage I/II melanomas, 4 dysplastic nevi, 10 melanocytic nevi and 4 patients before and after BRAF inhibitor treatment	M14, LOX IMVI, COLO 38, MALME-3M, SKMEL5, WM115, WM266, M229, HEK-293	Yes	BRAF, MDM2	–	–	Inhibits growth and migration of melanoma cells, induces apoptosis and impairs drug resistance in melanoma	(243)
*miR-101*	–	HEK293T, NHEM, 29 melanoma cell lines established from metastases of melanoma patients	Yes	MITF, EZH2		Patient survival	Suppresses proliferation, migration and invasion of melanoma cells through targeting MITF and EZH2	(244)
*miR-664*	9 melanoma and 2 BMN tissues, 10 Nude mice (A375 cell line was injected to mice)	A375.S2, A7, MeWo, RPMI-7951, SK-MEL-5, SK-MEL-24, SK-MEL-28, PEM	Yes	PLP2	–	Patient survival	Decreases proliferation and tumorigenicity of melanoma cells through targeting PLP2	(245)
*miR-29a*	–	HACAT, HFF, A375, Malme-3M, SK-MEL-2, SK-MEL-5, M14	Yes	Bmi1	Wnt/β-catenin and NF-κB signaling pathways	–	Suppresses cell growth, migration and invasion in melanoma cells and induces apoptosis by targeting Bmi1	(246)
*miR-524-5p*	male NOD/SCID mice (SK-Mel-19 cell line was injected to mice)	HEK293, Malme-3M, Malme-3, A375, SK-Mel-19	Yes	BRAF, ERK2	MAPK/ERK signaling pathway	–	Inhibits proliferation and migration of melanoma cells through targeting BRAF and ERK2 and inhibition of MAPK/ERK signaling pathway	(247)
*miR-138*	Whole blood samples from 5 melanoma patients and 6 healthy controls	A2058	Yes	–	PI3K/AKT/mTOR signaling pathway	Patient survival	Suppresses cell proliferation and induces apoptosis in melanoma cells *via* inhibition of PI3K/AKT/mTOR signaling pathway	(248)
*miR-138*	–	WM451, HM	Yes	HIF-1α	–	–	Inhibits melanoma cells proliferation, invasion and glycolysis through targeting HIF-1α	(249)
*miR-138*	16 melanoma tissues and 16 precancerous tissues, female mice (WM35 and A375 cell lines were injected to mice)	WM35, A375, HEK293	Yes	HIF1α	–	–	Inhibits proliferation, migration and invasion and promotes apoptosis in melanoma cells through targeting HIF1α	(250)
*miR-126*	108 primary cutaneous melanoma tissues, 18 melanoma metastases and 16 dysplastic nevi samples	–	No	–	–	Patient survival, Breslow thickness, tumor ulceration, tumor stage	Can be an independent prognostic factor for overall survival	(251)
miR-126&126*	adult athymic nude mice (A375M and Me665/1 cell lines were injected to mice)	A375M, A375, Me665/1, NHEM, Me1007, Mel501, WM983A, Me1402/R, Me665/2, GR-mel, ST-mel,	Yes	ADAM9, MMP7	–	–	Decrease proliferation, invasion and chemotaxis of melanoma cells through targeting ADAM9 and MMP7	(252)
*miR-377*	FFPE tissues samples of 6 primary cutaneous melanoma and 13 benign nevi	mel33B1, mel-14PA, mel-15AY, mel-526, mel-624, NHEM	Yes	E2F3, MAP3K7	MAP3K7/NF-kB signaling pathway	–	Decreases proliferative ability and colony-forming capability in melanoma cells	(253)
*miR-139-5p*	82 malignant melanoma tissues and 30 benign skin disease tissues from healthy controls	PIG1, A375, SK-MEL-1, SKMEL-2, SK-MEL-5, SK-MEL-28	Yes	IGF1R	PI3K/AKT signaling pathway	–	Inhibits cell proliferation, migration and invasion and promotes apoptosis through targeting IGF1R	(254)
*miR-342*	27 melanoma tissues and paired ANTs	HEM, A375, A2058, SK-MEL-28, HT144	Yes	ZEB1	–	–	Inhibits proliferation and invasion of melanoma cells through targeting ZEB1	(255)
*miR-127*	40 melanoma tissues and paired ANTs, male BALB/c nude mice (WM35 cell line was injected to mice)	WM35, SK-MEL-5, SK-MEL-2, A375, HeMa-Lp	Yes	DLK1	–	Overall survival, tumor thickness, tumor stage	Suppresses cell proliferation and induces apoptosis through downregulation of DLK1	(256)
*miR-22*	48 melanoma tissues and paired ANTs, nude mice (A375 cell line was injected to mice)	HEM, A375, SK-MEL-1, WM35, SK-MEL-28	Yes	FMNL2	Wnt/β-Catenin Signaling Pathway	Overall survival, tumor thickness, TNM stage	Suppresses proliferation, migration and invasion of melanoma cells through targeting FMNL2	(257)
*miR-3065-5p*	12 primary melanoma and 9 benign melanocytic tumors	BRO, SK-MEL1	Yes	HIPK1, ITGA1	–	–	Induces cell cycle arrest at G1 phases and inhibits migration of melanoma cells	(258)
*miR-204-5p*	12 primary melanoma and 9 benign melanocytic tumors	BRO, SK-MEL1	Yes	–	–	–	Decreases proliferation, invasion and colony formation ability of melanoma cells	(258)
*miR-204-5p*	30 melanoma tissues and 20 benign nevi tissues, 10 immunodeficient female nude mice (A375 cell line was injected to mice)	A375, WM35, SK-MEL-5, SK-MEL-2	Yes	MMP9, BCL2	–	Overall survival	Inhibits proliferation, migration and invasion and induces apoptosis in melanoma cells through targeting MMP9 and BCL2	(259)
*miR-610*	105 melanoma tissues and ANTs, female BALB/c mice (A375 and MV3 were injected to mice)	SK-MEL-1, A375, SK-MEL-28, MV3, B16-F1, HPM	Yes	LRP6	–	Patient survival, tumor stage, tumor thickness	Represses cell proliferation, cell cycle progression and induces apoptosis in melanoma cells by targeting LRP6	(260)
*miR-3662*	80 melanoma tissues and paired ANTs, BALB/c nude mice (A375 and OCM-1A cell lines were injected to mice)	A375, OCM-1A	Yes	ZEB1	–	–	Inhibits invasion and EMT process in melanoma cell *via* targeting ZEB1	(261)
*miR-331*	22 melanoma tissues and paired ANTs	HEM, A375, A2058, HT144, SK-MEL-1, SK-MEL-28	Yes	AEG-1	PTEN/AKT signaling pathway	–	Suppresses proliferation and invasion of melanoma cells through targeting AEG-1	(262)
*miR-149-5p*	Melanoma tissues and ANTs	A2058, A375, HSC-1, SK-37, SKMLE-1, WM451, HaCaT	Yes	LRIG2	–	–	Reduces proliferation, colony formation and induces apoptosis in melanoma cells through targeting LRIG2	(263)
*miR-338-3p*	60 melanoma tissues and paired ANTs,	A375, G361	Yes	MACC1	–	clinical stage, lymph node metastasis	Inhibits proliferation, migration and invasion in melanoma cells through targeting MACC1	(264)
*miR-4458*	–	A375, A2058, SK-MEL-28, SK-MEL-2, HEMa-LP	Yes	PBX3	–	–	Represses proliferation, migration and induces apoptosis in melanoma cells *via* targeting PBX3	(265)
*miR-489-3p*	nude mice (A375 cell line was injected to mice)	A375, SK-MEL-2	Yes	SIX1	–	–	Inhibits proliferation, migration and invasion of melanoma cells and regulates glycolysis through targeting SIX1	(266)
*miR-431*	113 melanoma tissues and paired ANTs	A875, HBL, 1205Lu, A375, SK-MEL-1, HEMa-LP, CHL-1	Yes	NOTCH2	–	Overall survival, tumor stage, ulceration	Suppresses cell proliferation, migration and invasion and induces apoptosis in melanoma cells through targeting NOTCH2	(267)
*miR-134*	18 melanoma tissues and paired ANTs	BT549, MB-231, MB-486, MCF7, SK-BR-3, 293T	Yes	CTHCR1	–	–	Decreases proliferation, migration, invasion and induces cell cycle arrest and apoptosis in melanoma cells through downregulation of CTHCR1	(268)
*miR-224-5p*	30 uveal melanoma tissues and paired ANTs	OCM‐1A, HEK 293T	Yes	PIK3R3, AKT3	–	–	Suppresses proliferation, migration and invasion of melanoma cells *via* targeting PIK3R3 and AKT3	(269)
*miR-140-5p*	25 melanoma tissues and paired ANTs, 20 adult athymic nude mice (SK‐MEL‐1 cell line was injected to mice)	A375, A875, SK‐MEL‐5, SK‐MEL‐1, SK‐MEL‐28, HEMa‐LP, HaCaT	Yes	SOX4	Wnt/β‐catenin and NF‐κB signaling pathways	clinical stage	Its overexpression inhibits proliferation and invasion of melanoma cells by targeting SOX4 and inactivation of Wnt/β-catenin and NF‐κB signaling pathways	(270)
*miR-140-3p*	25 melanoma tissues and paired ANTs, 30 male BALB/c nude mice (M229, A375 and M14 cell lines were injected to mice)	M14, MALME-3M, M229, WM226, A375, SKMEL5, LOX IMVI, HPM	Yes	ABHD2	JNK and AKT/p70S6K Signaling Pathway	Overall survival	Blocks proliferation, migration and invasion and induces apoptosis in melanoma cell through targeting ABHD2	(271)
*miR-135b*	27 melanoma tissues and 27 normal skin tissues	A375, PEM	Yes	RBX1	–	–	Suppresses proliferation, migration and invasion of melanoma cells through targeting RBX1	(272)

## Diagnostic/prognostic miRNAs in melanoma

Hanniford et al. have introduced a miRNA panel consisting of miR-150-5p, miR-15b-5p, miR-16-5p, and miR-374b-3p whose expression levels could predict the possibility of brain metastasis of melanoma tumors along with clinical stage. Moreover, Kaplan-Meier analysis showed the significance of this miRNA panel in determination of brain-metastasis-free and overall survival of patients with melanoma (273). Stark et al. have assessed expression levels of 17 miRNAs in both melanoma tissues and serum samples of these patients compared with cancer-free individuals. Expression levels of these miRNAs in melanoma samples have been shown to predict stage, recurrence, and survival of patients. Notably, serum expression of a seven-miRNA panel could distinguish melanoma patients from control subjects with 93% sensitivity and more than 82% specificity if at least 4 miRNAs were expressed. Based on the superiority of this miRNA panel above the conventional serological biomarkers for melanoma, it has been suggested as a tool for monitoring disease course in early metastatic melanoma cases to identify relapse after tumor excision or adjuvant therapy (23). Worley et al. have used a high throughput technique to identify the miRNAs whose expression profile could predict the metastatic potential of uveal melanomas. Their approach led to identification of let-7b and miR-199a as the most robust discriminators. Notably, expression profile of six miRNAs could differentiate low and high risk groups with optimal sensitivity and specificity values (274). **Table 3** shows the role of miRNAs in the prediction of prognosis of melanoma using Kaplan-Meier or Cox regression analyses.

**Table 3:** Role of melanoma in prediction of prognosis of melanoma (DMFS, distant metastasis free survival; OS, overall survival; DFS, disease-free survival; RFS, relapse-free survival; MSS, melanoma specific survival).

microRNA	Sample number	Kaplan-Meier analysis	Univariate cox regression	Multivariate cox regression	Reference
*miR-10b*	79 primary melanoma tissues and 32 metastases	–	Is a potential prognostic biomarker associated with metastasis	Can be an independent potential prognostic factor	(33)
*miR-10b*	78 melanoma tissues and 30 non-tumor skin samples	Its high expression is associated with poor OS in melanoma patients.	–	–	(35)
*miR-10b*	Blood samples from 85 melanoma patients and 30 healthy volunteers	Its high serum levels is associated with short DFS and OS.	–	Its serum level is an independent prognostic factor for OS and CFS in melanoma patients.	(275)
*miR-30d*	109 primary melanoma tissues and 17 melanoma metastases	Its high expression is associated with poor OS.	–	Its expression pattern is an independent prognostic factor for melanoma mortality.	(65)
*miR-30b*	109 primary melanoma tissues and 17 melanoma metastases	Its high expression is associated with poor OS.	–	–	(65)
*miR-92a*	75 melanoma tissues and paired ANTs	Its high expression is associated with poor OS.	–	–	(75)
*miR-596*	36 melanomas samples and 22 nevi	Its low expression was associated with significantly shorter OS.	–	–	(126)
*miRNA-29c*	30 malignant melanoma tissues and 10 paracancer tissues	Its low expression associated with poor prognosis.	–	–	(128)
*miR-365*	40 melanoma tissues and paired ANTs	Its low expression associated with shorter OS and RFS.	–	–	(97)
*miR-137*	30 primary melanoma tissues and paired ANTs	Its low expression associated with poor survival.	–	Can be an independent risk factor of OS	(141)
*miR-137*	97 melanoma tissues and paired ANTs	Its low expression is associated shorter OS in melanoma patients.	–	Its expression is an independent prognostic marker of OS in melanoma patients.	(146)
*miR-142-3p* *miR-142-5p*	66 stage III FFPE tissues	Their low expression associated with poor survival.	–	–	(118)
*miR-21*	86 primary cutaneous melanomas tissues, 10 melanoma metastases, 10 dysplastic nevi samples	Its high expression associated with shorter 5-year DFS and shorter 5-year OS.	–	Its expression pattern can be an independent prognostic factor for overall survival in melanoma patients.	(37)
*miR-21*	12 FFPE primary melanoma tissues and 12 melanocytic nevi	Its high expression is associated with poor RFS and OS.	–	–	(38)
*miR-181*	17 matched melanoma tissues before and after resistance of patients to BRAF inhibitors	Its low expression is correlated with low progression free survival (PFS) and OS	–	–	(172)
*miR-4633-5p*	56 Primary human sinonasal mucosal melanoma tissues	–	Its expression pattern can be a prognostic factor in identifying metastatic sinonasal mucosal melanoma.	It can be an independent prognostic factor for metastasis.	(175)
*miR-191*	32 lymph node metastases	Its low expression associated with poor melanoma-specific survival.	–	–	(276)
*miR-193b*	32 lymph node metastases	Its high associated with poor melanoma-specific survival.	–	–	(276)
*hsa-miR-211-5p* *hsa-miR-514a-3p* *hsa-miR-508-3p* *hsa-miR-509-3-5p* *hsa-miR-513c-5p* *hsa-miR-513a-5p*	UM dataset of miRNA expression profiles was obtained from the UCSC Xena Browser	Their high expression were associated with poor OS.	–	–	(277)
*hsa-let-7b-5p* *hsa-miR-452-5p* *hsa-miR-224-5p* *hsa-miR-592* *hsa-let-7b-3p* *hsa-miR-199a-5p*		Their low expressions were associated with poor OS.	–	–	(277)
*Six miRNAs signature:* *mir-15* *mir-342-3p* *mir-455-3p* *mir-145* *mir-155* *mir-497*	59 metastatic and primary melanoma, Congenital nevi	This signature can estimate post-recurrence survival.	–	This miRNA signature is an independent predictor of post-recurrence survival in metastatic melanoma.	(278)
*miR-338-5p*	46 melanoma tissues and 25 normal nevi samples	Its high expression is associated with decreased OS.	Its expression correlates to patient survival	It can be an independent prognostic factor for OS.	(48)
*miR-203*	148 melanoma tissues and paired ANTs	Its low expression is associated with poor OS.	–	It can be an independent prognostic marker for melanoma patients.	(136)
*miR-29c*	149 melanoma tissues with AJCC stage I–IV	Its low expression is associated with poor DFS and OS in stage III melanoma patients.	Its expression correlates to DFS and OS	Its expression is significantly correlated to OS but not DFS.	(279)
*miR-206*	serum samples from 60 melanoma patients and 30 healthy controls	Its low serum levels is associated with poor DFS and OS.	–	Its serum level is independent prognostic factors for DFS and OS.	(185)
*miR-18b*	92 primary melanoma tissues and 48 benign nevi samples	Its low expression is associated with shorter OS.	–	–	(199)
*miR-15b*	128 FFPE tissues of primary melanomas and 11 melanocytic nevi samples	Its high expression is associated with RFS and OS.	–	Its expression pattern can be an independent prognostic factor for DFS and OS.	(50)
*miR-183*	30 melanoma tissues and 14 normal skin samples	Its low expression is associated with poor OS.	–	–	(214)
*miR-23a*	Serum samples from 192 melanoma patients and 51 matched cancer-free controls	Its low serum level is associated with poor OS.	Its serum level is predictor of patients OS.	Its serum level is an independent prognostic biomarker for OS.	(106)
*miR-23b*	114 primary melanoma tissues and ANTs	Its low expression is associated with short 3-year survival in melanoma patients.	–	–	(107)
*miR-216a-5p*	86 uveal melanoma tissues	Its low expression is associated with poor DFS and OS.	–	–	(115)
*miR-221*	Serum samples from 72 cutaneous malignant melanoma and 54 healthy controls	Its high expression is associated with poor RFS and OS.	–	Its expression can be an independent predictor of DFS and OS	(55)
*miR-205*	319 melanoma tissue samples	Its low expression is associated with short MMS in melanoma patients.	Its expression can be a predictor of MMS	Its expression pattern can be an independent prognostic marker for MMS	(280)
*miR-205*	65 primary melanoma tissues and 67 melanoma metastases	Its low expression is associated with shorter DMFS and MSS.	–	Its expression pattern can be an independent prognostic factor of MMS	(125)
*miR-200c*	65 primary melanoma tissues and 67 melanoma metastases	Its low expression is associated with shorter DMFS and MSS.	–	Its expression pattern can be an independent prognostic factor of survival	(125)
*miR-200a*	46 melanoma tissues and paired ANTs	Its low expression is associated with poor OS.	–	–	(195)
*miR-125*	65 primary melanoma tissues and 67 melanoma metastases	Its low expression is associated with shorter DMFS and MSS.	–	Its expression pattern can be an independent prognostic factor of survival	(125)
*miR-150*	51 melanoma tissues and paired ANTs	Its low expression is associated with short RFS and OS.	–	–	(117)
*let-7b*	106 mucosal melanoma tissues, mucosal nevi samples	Its low expression is associated with poor DFS.	Its expression level correlates with DFS in melanoma patients	Its expression pattern is an independent prognostic marker for DFS	(211)
*let-7c*	106 mucosal melanoma tissues, mucosal nevi samples	Its low expression is associated with poor DFS.	Its expression level correlates with DFS in melanoma patients	Its expression pattern is an independent prognostic marker for DFS	(211)
*miR-126*	108 primary cutaneous melanoma tissues, 18 melanoma metastases and 16 dysplastic nevi samples	Its low expression is associated with poor OS in melanoma patients	–	Can be an independent prognostic factor for overall survival	(251)
*miR-127*	40 melanoma tissues and paired ANTs	Its low expression is associated with short OS	–	–	(256)
*miR-22*	48 melanoma tissues and paired ANTs	Its low expression is associated with shorter OS	–	–	(257)
*miR-610*	105 melanoma tissues and ANTs	Its low expression is associated with short 5-year survival	–	–	(260)
*miR-431*	113 melanoma tissues and paired ANTs	Its low expression is associated with poor OS in melanoma patients	Can be a potential prognostic marker for melanoma patients	Can be an independent prognostic factor for melanoma patients	(267)
*miR-140-3p*	25 melanoma tissues and paired ANTs	Its low is associated with poor OS	Its expression pattern correlates with OS in melanoma patients	Its expression pattern is an independent prognostic factor for OS in melanoma patients	(271)
*miR-125b*	29 FFPE melanoma specimens and 16 intradermal nevus specimens	Its low expression is associated with short OS	–	Its expression level can be an independent prognostic marker for OS	(281)

Receiver operating characteristic (ROC) curves have been used to assess the diagnostic or prognostic values of miRNAs in melanoma. Based on the area under curve (AUC) values, several miRNAs can be suggested as appropriate biomarkers for this kind of cancer. In the field of miRNA application in melanoma diagnosis, these curves depict the diagnostic capability of expression level of a miRNA as a binary classifier system for detection of melanoma cases as its discrimination threshold is changed. In other words, these curves are generated by plotting the true positive rate against the false positive rate at different threshold points. Notably, serum expression levels of several miRNAs have high sensitivity and specificity values for differentiating between melanoma patients and healthy subjects or between metastatic and non-metastatic melanomas. **Tables 4**, **5** list the miRNAs whose application as diagnostic or prognostic markers has been evaluated using ROC curve analysis, respectively.

**Table 4:** Application of miRNAs as diagnostic tools in melanoma.

microRNA	Expression pattern	Sample	Diagnostic biomarker	ROC curve analysis			Reference
				Sensitivity	Specificity	Area under the ROC curves (AUC)	
miR-16 miR-211-5p miR-4487 miR-4706 miR-4731 miR-509-3p miR-509-5p	Upregulated Upregulated Downregulated Downregulated Downregulated Downregulated Downregulated	Serum samples	Diagnostic (diagnosis of presence of melanoma)	93%	≥ 82%	–	(23)
miR-211-5p	Downregulated	Tissue samples	Diagnostic (invasive melanoma)	–	–	0.933	(282)
		Tissue samples	Diagnostic (melanoma in situ)	–	–	0.933
		Tissue samples	Diagnostic (dysplastic nevi)	–	–	0.951
miR-211	Downregulated	Tissue samples	Diagnostic (discriminating melanomas from nevi)	90%	86.2%	0.862	(283)
miR-532-5p miR-106b	- -	Serum exosomes	Diagnostic (distinguishing melanoma patients from healthy individuals)	–	–	0.936	(284)
miR-15b-5p	Upregulated	Plasma samples	Diagnostic (diagnosis of cutaneous melanoma)	90.0%	–	0.80	(285)
miR-150-5p	Upregulated	Plasma samples	Diagnostic (diagnosis of cutaneous melanoma)	96.7%	–	0.94
miR-149-3p	Upregulated	Plasma samples	Diagnostic (diagnosis of cutaneous melanoma)	93.3%	–	0.95
miR-193a-3p	Downregulated	Plasma samples	Diagnostic (diagnosis of cutaneous melanoma)	76.7	–	0.84
miR-524-5p	Downregulated	Plasma samples	Diagnostic (diagnosis of cutaneous melanoma)	90.0%	–	0.80
miR-149-3p miR-150-5p miR-193a-3p	Upregulated Upregulated Downregulated	Plasma samples	Diagnostic (diagnosis of cutaneous melanoma)	94.8%	–	0.97
hsa-miR-186 hsa-let-7d hsa-miR-18a hsa-miR-145 hsa-miR-99a hsa-miR-664 hsa-miR-501-5p hsa-miR-378 hsa-miR-29c hsa-miR-1280 hsa-miR-365 hsa-miR-1249 hsa-miR-328 hsa-miR-422a hsa-miR-30 d hsa-miR-17	Upregulated Upregulated Upregulated Upregulated Upregulated Upregulated Upregulated Upregulated - Upregulated Upregulated Upregulated Upregulated Upregulated - Downregulated	Blood samples (expression of miRNAs in blood cells)	Diagnostic	98.9%	95%	97.4%	(286)
miR-125b	Downregulated	Tissue samples	Diagnostic biomarker (diagnosis of melanoma)	–	–	0.880	(281)
miR-211	Downregulated	Serum samples	Diagnostic biomarker (distinguish metastatic from uveal melanoma localized one)	–	–	0.96	(287)
miR-16 miR-145 miR-146a miR-204 miR-211 miR-363-3p	Downregulated Downregulated Downregulated Downregulated Downregulated Downregulated	Serum samples	Diagnostic biomarker (identifying uveal melanoma)	93%	100%	–
miR-10b	Upregulated	Serum samples	Diagnostic biomarker (distinguishing melanoma patients from controls)	–	–	0.841	(275)

**Table 5:** Prognostic role of miRNAs in melanoma as identified by ROC curve analysis.

microRNA	Expression pattern	Sample	Prognostic biomarker	ROC curve analysis			Reference
				Sensitivity	Specificity	Area under the ROC curves (AUC)	
miR-150-5p	Downregulated	Serum samples	Prognostic (discrimination of survival in stage IV)	–	–	0.69	(118)
		Tissue samples	Prognostic (discrimination of stage)	–	–	0.733
miR-142-3p	Downregulated	Serum samples	Prognostic (discrimination of stage IV from stage III)	–	–	0.69
		Tissue samples	Prognostic (discrimination of stage)	–	–	0.797
miR-142-5p	Downregulated	Tissue samples	Prognostic (discrimination of stage)	–	–	0.733
miR-150-5p miR-142-3p miR-142-5p	Downregulated Downregulated Downregulated	Tissue samples	Prognostic (discrimination of stage)	–	–	0.838
miR-4633-5p	Downregulated	Tissue samples	Prognostic (identifying metastatic sinonasal mucosal melanoma)	87.5%	100%	0.88	(175)
miR-1246 miR-185	Upregulated Upregulated	Plasma samples	Prognostic (identifying metastatic melanoma)	90.5%	89.1%	–	(288)
miR-9 miR-145 miR-150 miR-155 miR-205	Upregulated - - - -	Serum samples	Prognostic (distinguishing metastatic melanoma)	–	–	0.77	(289)
miR-532-5p miR-106b	- -	Serum exosomes	Prognostic (distinguishing patients with and without metastasis)	–	–	0.818	(284)
		Serum exosomes	Prognostic (discriminates stage I–II patients from stage III–IV patients)	–	–	0.820
miR-23a	Downregulated	Serum samples	Prognostic (distinguishing primary melanoma from metastatic one)	76.0%	75.3%	0.797	(106)
miR-195 miR-224 miR-365a miR-365b miR-452 miR-4709 miR-7702 miR-513c miR-873	- - - - - - - - -	Tissue samples	Prognostic biomarker (for OS)	–	–	0.858	(290)
let-7b	Downregulated	Tissue samples	Prognostic biomarker (for DFS)	–	–	0.634	(211)
let-7c	Downregulated	Tissue samples	Prognostic biomarker (for DFS)	–	–	0.647
miR-10b	Upregulated	Serum samples	Prognostic biomarker (advanced stage vs early stage)	–	–	0.785	(275)

## Implications of miRNAs in the Treatment of Melanoma

miRNAs are implicated in the therapeutic effects of several anti-cancer agents. For instance, Genistein, the isoflavone extracted from soybean, has been shown to suppress proliferation of human uveal melanoma cells possibly through modulating expression of miR-27a and its target gene ZBTB10 (291).

miRNAs are also involved in conferring resistance to immunotherapeutic modalities. For instance, expression of miR-222 has been shown to be higher in melanoma samples obtained from patients who did not respond to ipilimumab compared with those benefitting from this option (292). Mechanistically, the ADAR1/miR-222/ICAM1 axis has been reported to be involved in this process (292). Other miRNAs such as miR-488-3p, miR-195 and miR-211 participate in the regulation of response to the chemotherapeutic agent cisplatin (130, 162, 163)

Application of miRNAs in the therapeutic settings is limited by target specificity issues (293). However, some miRNAs are currently being tested in some diseases. Among these therapeutic modalities are miR-122/miravirsen and miR-92/MRG 110 which have been manufactured by Roche/Santaris and Regulus Therapeutics, respectively (293).

## Association Between Polymorphisms Within miRNAs and Risk of Melanoma

Theoretically, polymorphisms with miRNA coding genes can alter their expression or function. Although such polymorphisms are predicted to influence the risk of different cancers such as melanoma, this field has not been vastly explored. Few studies have assessed association between a certain polymorphism within miR-146a namely the rs2910164 G/C and melanoma risk. In spite of the proposed role for allele C of this polymorphism in conferring risk of melanoma (294, 295), cell line studies have shown that G allele confers high proliferative capacity to melanoma cells (296). **Table 6** summarizes the results of these studies.

**Table 6:** Summary of studies which assessed association between miRNA polymorphisms and risk of melanoma.

microRNA	SNP	Genotyping method	Samples	Association with melanoma	Functional experiments	Reference
miR-146a	rs2910164G>C	–	Blood samples from 224 patients and 264 healthy controls	Allele C was associated with risk of melanoma in males and has allelic dosage effect (CC homozygotes has greater risk)	–	(294)
miR-146a and RNASEL polymorphisms interaction	rs2910164 G/C (in miR-146a)	PCR-RFLP	Blood samples from 304 sporadic melanoma patients and 314 control individuals	Men carrying allelic combination miR-146a rs2910164 C and RNASEL rs486907 A have highest risk of melanoma	–	(295)
	rs486907 A/G (in RNASEL)					
miR-146a	rs2910164 G/C	PCR-RFLP	Skin samples from 50 melanoma patients and 107 controls, 8 blood samples from patients	GC genotype was significantly increased in the patients compared with the controls	G allele confers high proliferative capacity to melanoma cell lines and GC cell lines have more invasive and migratory ability than CC cell lines	(296)

## Discussion

Dysregulation of miRNAs in melanoma samples and cell line have been reported by several studies. The functional consequences of such dysregulation on cell behavior have also been appraised. However, the underlying mechanism of such dysregulation is not clarified completely. Copy number variations in miRNA-coding genes or genes associated with the biogenesis or function of miRNAs may be responsible for the observed dysregulation of miRNAs in melanoma and other types of cancers (10). Moreover, the role of epigenetic factors in this process should not be ignored. For instance, CpG methylation of the miR-34a promoter has been suggested as an underlying mechanism for down-regulation of this miRNA in primary melanoma samples and melanoma cell lines (297). Another possible mediators of miRNA dysregulation in the melanoma are melanoma-inducing transcription factors such as MITF whose role in the expression of a number of miRNAs has been verified (298). As several miRNAs are implicated in the modulation of skin response to ultraviolet radiation (299), this environmental carcinogen might also affect expression of miRNAs which are involved in the melanomagenesis.

Mechanistically, several melanoma-associated miRNAs function upstream or downstream of known oncogenes in melanoma. For instance, miR-137 and miR-182 are among miRNAs that target MITF oncogene (54, 300). Moreover, expressions of several miRNAs such as a number of let-7 family members, miR-221/222, miR-17-92 and miR-106-363 clusters, miR-29, miR-146a, miR-148b, and miR-125b have been shown to be modulated by MITF (298). Moreover, several miRNAs such as miR-7, miR-23a and miR-596 have functional interactions with MAPK/ERK and PI3K/PTEN/Akt signaling pathways in the context of melanoma. A number of miRNAs such as miR-378, miR-10b, miR-25, miR-485-5p, miR-708, miR-136, miR-488-5p, miR-29a, miR-22 and miR-140-5p have interactions with Wnt/β-catenin pathway. Finally, miR-21, miR-7-5p, miR-23b, miR-145-5p, miR-9, miR-29a, miR-377 and miR-140-5p interacts with NF-κB signaling in the context of melanoma development. Thus, a number of miRNAs provide functional links between cancer-related pathways in this context.

miRNAs have functions both in the paternal cell in which they are produced as well as in the adjoining cells. These transcripts can modulate characteristics of adjacent melanoma cells or directly affect tumor niche by modifying extracellular matrix and function of resident cells in this environment including fibroblasts and endothelial or immune cells. This activity of miRNAs potentiates them as contributors of melanoma metastatic potential through affecting intravasation of cancer cells into vessels, viability of tumor cells in the circulation, their leakage in the target tissues, and establishment of the pre-metastatic milieu in remote organs (301).

Several miRNAs have been shown to differentiate melanoma patients from healthy subjects or distinguish between metastatic and non-metastatic melanoma patients. The prognostic assays founded on miRNAs signature can enhance the efficacy of conventional staging systems in predicting patients’ prognosis and their management in the clinical settings in the terms of choosing adjuvant therapies or clinical trial enrolment. Therefore, these miRNAs are potential biomarkers for this kind of skin cancer.

Numerous miRNAs have been dysregulated in tumor samples or peripheral blood of patients with melanoma. Such dysregulation can be used as biomarker for early detection of melanoma or follow-up of patients after initial treatments to uncover any possible tumor recurrence. Blood-based biomarkers are expected to substitute invasive methods of cancer diagnosis in future. Based on the heterogeneous pattern of miRNAs expression in tumor samples and the varied expressions among affected individuals, multi-miRNA panels are more promising in the diagnostic approaches compared with individual miRNAs.

Finally, miRNAs might be implicated in the anti-cancer effects of a number of therapeutic agents including both chemical and herbal medicines. Evidence for supporting this idea has come from several studies including a study which revealed the role of miR-27a in mediating the anti-proliferative effects of Genistein in human uveal melanoma cells (291). Moreover, the observed up-regulation of miR-222 in melanoma samples obtained from patients who did not respond to ipilimumab compared with those benefitting from this option (292) implies its contribution in resistance to this agent. Therefore, miRNAs are promising targets for modulation of response of melanoma cells to a wide range of therapeutic options.

## Perspectives and Future Directions

Assessment of expression pattern of miRNAs in cohorts of melanoma patients from different ethnicities and uncovering their association with genetic polymorphisms would facilitate design of prognostic/diagnostic panels. The relationship between aberrant miRNA profile and response to therapeutic regimens should be unraveled. Such kinds of approaches pave the way for design of personalized methods of treatment of melanoma. Therapeutic targeting of miRNAs can influence melanoma course and enhance sensitivity to both conventional therapies and immunotherapeutic approaches. Yet, safety and bioavailability issues remained to be solved before implementation of these techniques in the clinical settings.

## Author Contributions

SG-F and MT wrote the draft and revised it. MG collected the tables and designed it. All authors contributed to the article and approved the submitted version.

## Conflict of Interest

The authors declare that the research was conducted in the absence of any commercial or financial relationships that could be construed as a potential conflict of interest.
